# Fluorescence and Spectral Imaging

**DOI:** 10.1100/tsw.2007.308

**Published:** 2007-12-21

**Authors:** Ralph S. DaCosta, Brian C. Wilson, Norman E. Marcon

**Affiliations:** ^1^ Department of Medical Biophysics, University of Toronto, Ontario Cancer Institute, 610 University Avenue, Toronto, Ontario, M5G 2M9, Canada; ^2^ St. Michael's Hospital, Center for Therapeutic Endoscopy and Endoscopic Oncology, 16-062 Victoria Wing, 30 Bond Street, Toronto, Ontario, M5B 1W8, Canada

**Keywords:** Fluorescence, endoscopy, dysplasia, Barrett's esophagus, quantum dots, nanomedicine, optical, probes, early detection

## Abstract

Early identification of dysplasia remains a critical goal for diagnostic endoscopy since early discovery directly improves patient survival because it allows endoscopic or surgical intervention with disease localized without lymph node involvement. Clinical studies have successfully used tissue autofluorescence with conventional white light endoscopy and biopsy for detecting adenomatous colonic polyps, differentiating benign hyperplastic from adenomas with acceptable sensitivity and specificity. In Barrett's esophagus, the detection of dysplasia remains problematic because of background inflammation, whereas in the squamous esophagus, autofluorescence imaging appears to be more dependable. Point fluorescence spectroscopy, although playing a crucial role in the pioneering mechanistic development of fluorescence endoscopic imaging, does not seem to have a current function in endoscopy because of its nontargeted sampling and suboptimal sensitivity and specificity. Other point spectroscopic modalities, such as Raman spectroscopy and elastic light scattering, continue to be evaluated in clinical studies, but still suffer the significant disadvantages of being random and nonimaging. A recent addition to the fluorescence endoscopic imaging arsenal is the use of confocal fluorescence endomicroscopy, which provides real-time optical biopsy for the first time. To improve detection of dysplasia in the gastrointestinal tract, a new and exciting development has been the use of exogenous fluorescence contrast probes that specifically target a variety of disease-related cellular biomarkers using conventional fluorescent dyes and novel potent fluorescent nanocrystals (i.e., quantum dots). This is an area of great promise, but still in its infancy, and preclinical studies are currently under way.

## INTRODUCTION

Although there continues to be a major role of endoscopy in the investigation and diagnosis of patients with symptoms, there has been a major thrust around the world in the screening of asymptomatic patients who are at risk for a variety of malignancies related to age, family history, genetics, and areas of geographic origin. Although these risks are recognized and several algorithms have been devised by medical epidemiologists to assess a variety of complex issues, such as cost effectiveness, social strata, and health delivery, a major deficiency is the absence of suitable organ-specific biomarkers. Rather than proposing colon cancer screening and colonoscopy for all average-risk citizens over the age of 50, it would be an enormous benefit to have a reliable biomarker to better select patients for endoscopic investigation. Most endoscopists, providing they exercise reasonable care, should be able to identify cancers and most polyps that are at least greater than 5–10 mm in diameter. The challenge, even among experienced endoscopists, is to identify flat and not readily discernable lesions. Therefore, it should be the aim of all endoscopists to detect intramucosal cancers at the stage of dysplasia and before they become invasive. Training and discipline in visual recognition for these lesions is a fundamental requirement. We have learned a great deal from our Japanese colleagues on the importance of meticulous endoscopic examination. This difference between Japanese and Western endoscopists was aptly stated by Dr. Rene Lambert as the "*The Eyes Wide Shut*" concept[1]. Although scolding, this opinion raises the fact that it is the smaller and more troublesome subtle dysplastic lesion that presents the greatest challenge for the endoscopist. Detection of the flat dysplastic lesions remains problematic and most endoscopists would be greatly helped with a visual "crutch" to aid in identifying these occult, but clinically important, lesions. These optically based aids range from inexpensive "low-tech" sprayable vital and contrasting agents, as used in chromoendoscopy, to the more recent optically sophisticated and expensive technologies, such as narrow band imaging, autofluorescence imaging, optical coherence tomography, and confocal fluorescence microendoscopy. Where these newer technologies fit into the modern diagnostic algorithm continues to be a source of ongoing research and debate. None of these alternative endoscopic technologies have been subjected to rigorous large-scale prospective multicenter trials to assess their true clinical impact. Nonetheless, there is a crucial void to be filled and these new devices, while having great promise, remain investigational.

With a broad or wide scan of an area of interest (i.e., the colonic lumen, stomach, or the esophagus affected by Barrett's), the endoscopist is searching for a "red flag" or "waving hand" that focuses his attention on a suspicious area that may or may not have physical irregularities (i.e., a plaque or nodule), but may be dysplastic. This area of interest will then be more closely examined and with current practice will have targeted biopsies, as is current practice. A speculation of the future may be the role of "optical biopsy" (i.e., confocal fluorescence microendoscopy, optical coherence tomography, or endocytoscopy), which may offer a real-time *in situ* histological-grade diagnosis. Optical biopsy will only be useful once a lesion has been identified and requires further histological definition, and as such will never be a primary screening tool. Narrow band imaging and autofluorescence endoscopy, or conventional dye spraying, have the potential to survey wide areas of the mucosa looking for occult dysplasia or more clearly defining recognizable nodules and plaque-like lesions.

The goal of this article is to elaborate on the mechanisms and current state of the art of tissue autofluorescent endoscopic imaging.

## Tissue Autofluorescence (Light-Tissue Interactions)

To date, tissue fluorescence was one of the best-developed methods of enhancing conventional endoscopic diagnosis of gastrointestinal (GI) lesions. Tissues illuminated with ultraviolet (UV) or short wavelength visible (VIS) light from a laser or filtered light source emit fluorescence light of a longer wavelength (Fig. 1). Since the biological sources of this fluorescence are endogenous to the tissue, this type of tissue fluorescence is called "autofluorescence". The molecules responsible for this are called fluorophores and include such biological substances as connective matrix (collagen, elastin), cellular metabolic coenzymes (reduced nicotinamide adenine dinucleotide [NADH], flavin adenine dinucleotide ([FAD], and flavin mononucleotide [FMN]), aromatic amino acids (tryptophan, tyrosine, phenylalanine), byproducts of the heme biosynthetic pathway (porphyrins), and lipopigments (lipofuscin, ceroids). Each group of fluorophores is characterized by distinct excitation and emission wavelength ranges. It is common for a single excitation wavelength to excite many fluorophores and, conversely, for the emission

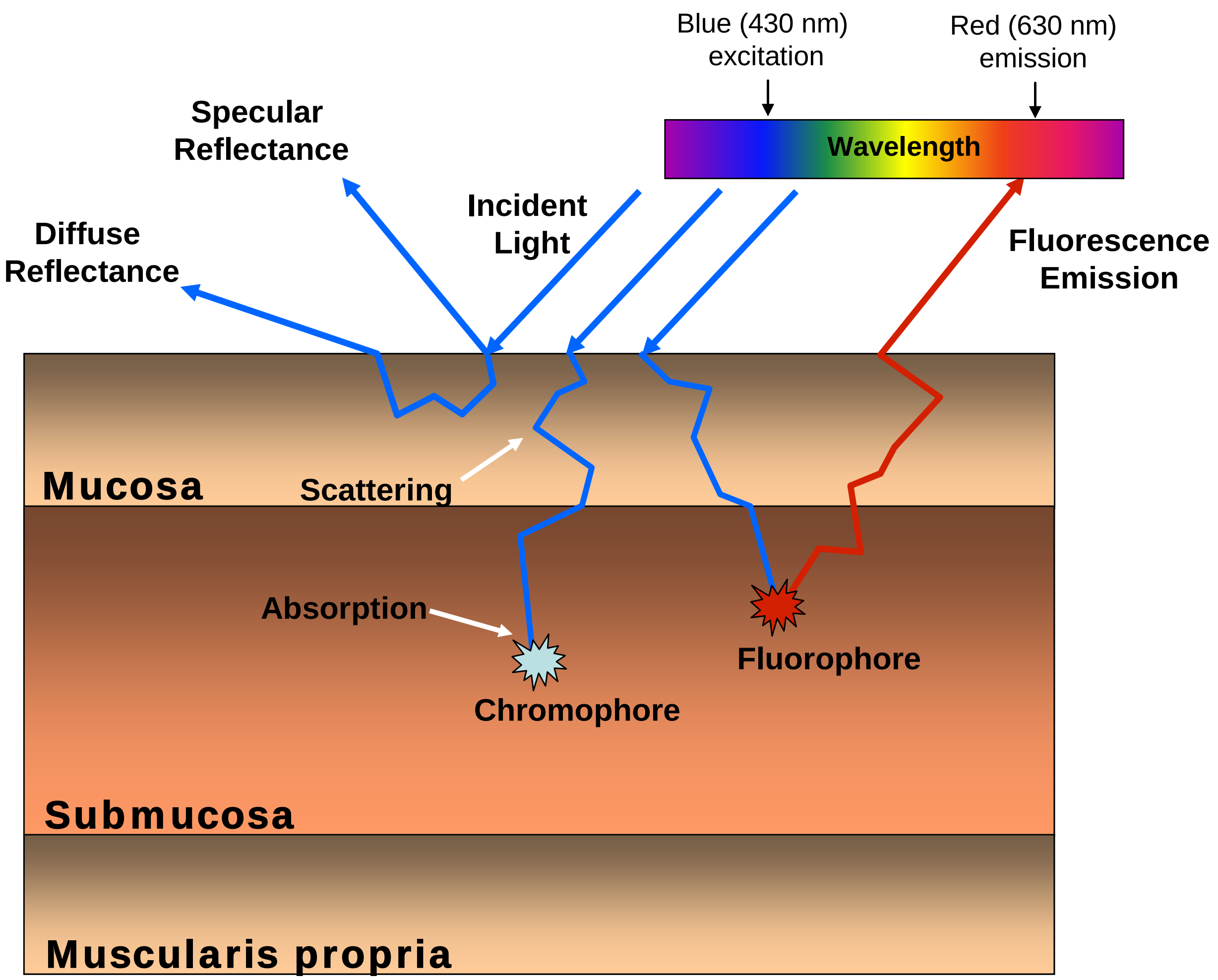

**FIGURE 1**. Schematic diagram illustrating various light-tissue interactions.

signals of many fluorophores to overlap, since the absorption and emission bandwidths of these molecules can be broad. Tissues also contain molecules called chromophores that absorb light without re-emission of fluorescence. The absorption is strongly wavelength dependent and may significantly modify the *in vivo* fluorescence spectrum observed at the tissue surface. The main chromophore in GI tissues in the visible wavelength (400–700 nm) range is hemoglobin.

While each fluorophore has a distinct fluorescence spectrum, tissues have a mixture of several fluorophores that occur in different concentrations and at different depths. Thus, the mucosa, submucosa, and muscularis propria have distinct fluorophore compositions, so that the fluorescence measured at the luminal surface comprises contributions from the fluorophores in the various layers. Since the excitation and fluorescence emission bands are often broad, relatively featureless, and overlap with one another, identifying individual fluorophores in a given tissue spectrum is difficult. Changes in the intrinsic fluorescence of the tissue layers with disease are due to alterations in their biochemical composition (metabolic state, microenvironment). In addition, changes in the layer thicknesses or in the blood content contribute to the fluorescence differences seen between normal and diseased tissues. Such changes collectively result in modifications to the intrinsic fluorescence of the tissue layers[2,3,4,5]. These fluorescence differences may be sufficient to distinguish between normal and diseased tissues. Thus, the use of autofluorescence to detect early cancers or premalignant colonic lesions is dependent on changes in one or more of the following factors: (a) the tissue architecture (mucosal thickening or loss of layered structures); (b) the light absorption and scattering properties of each layer, particularly hemoglobin in the capillary networks; (c) the distribution and concentration of fluorophores in the different layers; (d) the biochemical microenvironment of the tissue, which may alter the fluorescence yield or spectral shape; and (e) the metabolic status of the tissue (for example, changes in NADH fluorescence have been used to differentiate normal from dysplastic tissues[6]. Therefore, tissue autofluorescence is sensitive to alterations in tissue morphology, neovascularity, and biochemistry resulting from malignant transformation, although the details are complex. The degree to which disease changes *in vivo* fluorescence measurements is significantly wavelength dependent, since the excitation and emission wavelengths used determine the dominant fluorophores involved. For example, tissue proteins, composed of amino acids, are autofluorescent only when excited by UV wavelengths, while all other fluorophores (mentioned above) are excited by visible wavelengths. In general, as the wavelength increases, so too does the penetration depth of the excitation light. Thus, there may be one or more optimal excitation or emission wavelength bands, depending on the anatomical site or application. Unfortunately, for each type of tissue, the optimal excitation and emission wavelengths are not known *a priori* and must be determined from *ex vivo* tissue samples. This has the advantage of providing detailed fluorescence spectroscopy and microscopy, but the accuracy of the results must be considered carefully since the measured spectra may be altered by loss of blood or metabolic changes[2]. The measurement of excitation and emission matrices (EEMs) of known and suspected biological fluorophores *in solu* may also help to identify the optimal excitation and emission wavelength bands for autofluorescence spectroscopy and imaging of GI tissues[7].

## EXOGENOUS FLUORESCENT DRUGS

As an alternative to fluorescence endoscopic diagnosis based on tissue autofluorescence, exogenous fluorescent drugs (or prodrugs) that induce tissue fluorescence have been evaluated. Up to now, the majority of clinical reports have been based on the use of photosensitizers, some of which are inherently fluorescent and selectively localize within neoplastic tissues, for photodynamic therapy (PDT). Examples include hematoporphyrin derivative (HpD), benzoporphyrin derivative (BPD), tetra(m-hydroxyphenyl)chlorin (mTHPC), phthalocyanines (PC), and tin etiopurpurin ($SnET_2$). With drug-induced fluorescence, the fluorescent signal is strong compared to “background” tissue autofluorescence, resulting in an improved image contrast between tumor and surrounding normal tissue. Additionally, since the optimum excitation and emission wavelengths are known *a priori,* “background” tissue autofluorescence can be subtracted. However, despite these advantages, the diagnostic effectiveness of drug-induced fluorescence is dependent on the degree of selective localization of the drug in premalignant lesions. As well, such procedures must be performed at the optimum time following the administration of the drug in order for maximum tumor selectivity based on drug pharmacokinetics, which may vary from patient to patient and tissue to tissue. Although much lower fluorescent drug doses are needed for diagnosis than are typically used therapeutically, the use of such drugs is associated with extra costs, regulatory issues, and, in some cases, concern may be cutaneous photosensitization. The latter requires patients to limit severely their exposure to room and sun light for extended periods.

One compound that has shown promise to obtain good fluorescence contrast between normal and neoplastic tissues is 5-aminolevulinic acid (ALA). It is well established in many *in vitro* and *in vivo* systems that administering ALA in excess results in elevated endogenous synthesis of protoporphyrin IX (PpIX) via the heme biosynthesis pathway[8,9]. PpIX is a fluorescent photosensitizer that has a distinct red fluorescence emission between 625 and 725 nm. Since the PpIX is endogenously generated, there is often a high degree of tissue specificity, with particular localization in the mucosal layer of internal hollow organs, such as the lung[10], bladder[11], and GI tract[12]. Although not well understood, in general, PpIX appears to be generated in or accumulate preferentially in dysplastic tissues. However, contradictory results have been reported in *in vitro* studies of the differential synthesis of ALA-induced PpIX in tumor and normal epithelial cells[13]. Moreover, *in vivo* results have demonstrated significant variation in PpIX fluorescence from lesion to lesion in the same patient and a strong dependence on the anatomical site[9,12]. Imbalances in concentrations of endogenous iron, porphobilinogen deaminase, or in ferrochelatase, an enzyme involved in conversion of PpIX to heme, are considered possible reasons for increased tumor cell production of PpIX[14]. ALA has shown rapid metabolic breakdown[15], with few side effects, and a short duration of skin photosensitivity[16]. The chemical modification of ALA (e.g., ALA esters) has demonstrated some promise in increasing tissue penetration[17]. Additionally, administration of exogenous compounds, such as desferrioxamine[18], an iron chelator that inhibits conversion of PpIX to heme by ferrochelatase, may enhance PpIX accumulation, thereby allowing lower ALA doses. However, the optimal ALA dose and time delay before fluorescence detection remains at issue[19].

## CLINICAL EXPERIENCE OF TISSUE AUTOFLUORESCENCE

Point measurement techniques were the first to be used *in vivo* to detect tissue autofluorescence during GI endoscopy by collecting a spectral signal from the tissue surface. This early work then led to the development of *in vivo* fluorescence endoscopic imaging. Typically several meters long, a fiber optic probe used for fluorescence point spectroscopy can be composed of either single or multiple fibers. The probe is placed down the biopsy channel of the endoscope and in gentle contact with the tissue surface under white light endoscopy (WLE) control, it delivers excitation light (from a laser or wavelength filtered lamp) to the tissue surface and collects the resulting fluorescent light. Point fluorescence measurements are guided by white light viewing of the GI surface, but to prevent saturation of the fluorescence signal, these measurements must be performed either while the white light is temporarily turned off, by using a combination of pulsed excitation sources and fast-gated detectors, or by measuring the alternating-current fluorescence signal using an intensity-modulated excitation light source.

Typically, such probes (~0.5–1 mm in diameter) have a central delivery fiber that illuminates the tissue surface and a surrounding circular array of detector fibers that collect the emitted tissue fluorescence. Special optical filters block any scattered excitation light. In point measurements, the intensities of the component colors comprising the fluorescence signal are displayed as a function of wavelength, after separation in a spectrograph. The potential diagnostic value of these spectral data is then evaluated using a variety of mathematical procedures, such as taking the ratio of two or more fluorescence emission wavelength bands or analyzing the variance in spectral features. Recent reports have demonstrated the need for separate diagnostic algorithms for different segments of the GI tract[20,21,22,23].

Point spectroscopic measurements of tissue autofluorescence were the first approach used for diagnosis of the GI tract and initial studies were performed on *ex vivo* tissue samples. Early work involved the use of UV excitation wavelengths to differentiate between normal colonic mucosa, hyperplastic polyps, and dysplastic polyps (350–600 nm). For example, using 325-nm excitation, Kapadia et al.[20] applied multivariate linear regression analysis to the tissue fluorescence emission band, and demonstrated accuracies of 100, 94, and 100% for identifying normal, hyperplastic, and adenomatous tissues, respectively. By using a range of excitation and emission wavelengths, Richards-Kortum et al.[21] measured EEMs in *ex vivo* human colonic polyps, identifying 330, 370, and 430 nm as the optimal excitation wavelengths and 404, 480, and 680 nm as the optimal emission wavelengths (using 370-nm excitation) for accurate discrimination of adenomatous polyps from normal colon. Fluorescence spectroscopy was shown by Schomacker et al.[22] to differentiate between non-neoplastic and neoplastic colon tissues with a sensitivity and specificity of 80 and 92%, respectively.

Cothren et al.[5] were the first to report in an *in vivo* study using 370-nm excitation light and a 2D scatter plot of resulting fluorescence intensities at 460 and 680 nm for colonic tissues. This allowed adenomas and nonadenomatous colon tissue to be differentiated in 97% of cases. The first blinded *in vivo* fluorescence spectroscopy study of the colon reported by this group identified the correct tissue type in 88% of cases, with a sensitivity of 90% and a specificity of 95%[23].

For esophagus, the first *in vivo* fluorescence spectroscopy study was reported in 32 patients by Panjehpour et al.[24]. The optimal fluorescence emission wavelengths to differentiate normal from malignant tissues were determined using 410-nm excitation. Classification of resulting tissue fluorescence spectra was performed with a stepwise multivariate discriminant analysis. Normal vs. malignant tissues were classified with a sensitivity of 100% and a specificity of 98%.

In 1995, Vo-Dinh et al. reported the use of new diagnostic parameters based on the differential normalized fluorescence (DNF) signals for malignant esophageal tumor diagnosis[92]. Two hundred *in situ* measurements of endogenous fluorescence from normal and malignant esophageal tissues were performed during routine endoscopy in 48 patients using 410-nm excitation. Fluorescence spectra were analyzed using a method based on the emission intensity at 480 nm compared with the fluorescence signal integrated over all wavelengths, which yielded the DNF index. Briefly, the fluorescence emission spectra collected between 430 and 716 nm from 15 patients with normal squamous esophagus were normalized to the same total integrated intensity. This produced an average normalized fluorescence spectrum that was used as a baseline. By subtracting this baseline spectrum from each newly measured fluorescence spectrum, the DNF spectrum was determined. In this preliminary study, the DNF approach was compared with endoscopic examination and histopathology of biopsy samples. Excellent correlation in the classification of normal and malignant tumors for the samples was found, suggesting the potential of the DNF method to provide a direct, real-time, and *in situ* technique for esophageal cancer diagnosis.

In a follow-up study of 36 Barrett's patients, DNF index values at 480 nm for normal and cancerous tissues were selected and displayed as a scatter plot[25]. From the scatter plot, two populations representing normal and malignant DNF indices were separated with an accuracy of 96% of nondysplastic Barrett's esophagus, 100% for low-grade dysplasia (LGD)(classified as benign), and 90% for high-grade dysplasia (HGD)(classified as premalignant). However, 72% of LGD-containing focal HGD lesions were incorrectly identified as benign and in the same study, 7/7 patients with HGD, but 0/6 patients with LGD, were identified, thereby illustrating that this system could not detect low-grade lesions or focal HGD. To date, the utility of the DNF algorithm for fluorescence spectroscopic classification has been limited to the esophagus. Despite shortcomings, this important study demonstrated the potential to identify endoscopically occult premalignant lesions by autofluorescence point spectroscopy, thereby facilitating targeted biopsy.

Bourg-Heckly et al.[26] used 330-nm excitation in 24 patients with Barrett's esophagus. Three fluorescence emission ratios, $I_{390nm}/I_{450nm}$, $I_{550nm}/I_{450nm}$, and $I_{390nm}/I_{550nm}$, were used to differentiate each of normal, Barrett's esophagus, HGD, and carcinoma. They found that normal esophageal mucosa and specialized columnar Barrett's mucosa were similar, and that changes in spectral shape associated with neoplastic transformation were greater than intra- and interpatient spectral variations. Using $I_{390nm}/I_{550nm}$, the spectroscopy-based diagnosis differentiated neoplastic tissue from normal esophageal mucosa and specialized columnar Barrett's mucosa with a sensitivity and specificity of 86 and 95%, respectively.

Most of the *in vivo* point spectroscopy studies to date have involved steady-state fluorescence measurements. An alternative approach based on time-resolved autofluorescence spectroscopy was demonstrated recently when Mycek et al. reported the *in vivo* results of 17 patients with 24 polyps (13 adenomatous, 11 nonadenomatous)[27]. Time-resolved fluorescence spectra represent the decay of fluorescence intensity at a given emission wavelength as a function of time after a brief pulse of excitation light (~10 nsec). The decay time of colonic adenomas was shorter than that of nonadenomas, yielding an 85% differential sensitivity, 91% specificity, 92% positive predictive value, and 83% negative predictive value. Further evaluations are required to confirm the clinical effectiveness of this technique in other anatomic sites.

In an *in vivo* study involving 37 patients with Barrett's esophagus, Pfefer et al. evaluated the potential of using temporal and spectral fluorescence spectroscopy to improve the surveillance of HGD in Barrett's esophagus[28]. Briefly, 337- and 400-nm excitation wavelengths were used to acquire fluorescence spectra and transient decay profiles (at 550 ± 20 nm). Diagnostic algorithms were developed retrospectively using linear discriminant analysis based on spectral data to separate low-risk tissue (i.e., nondysplastic Barrett's esophagus, indefinite for dysplasia, and LGD) from HGD. Moderate levels of sensitivity and specificity were obtained for both 337 nm (sensitivity of 74%, specificity of 67%) and 400 nm (sensitivity of 74%, specificity of 85%) excitation. This study concluded that in the diagnosis of HGD in Barrett's esophagus, steady-state fluorescence was more effective than time-resolved fluorescence data, excitation at 400-nm excitation was more effective than 337 nm, and that sensitivity to dysplasia based on fluorescence-targeted biopsy remains suboptimal.

Unlike many point spectroscopy devices with single excitation wavelengths to produce tissue fluorescence, Georgakoudi et al. have made use of several excitation wavelengths and combined the resulting multiple fluorescence emission spectra and reflectance in assessing 16 patients with known Barrett's esophagus undergoing standard WLE surveillance[29]. Their prototype instrument used 11 different excitation wavelengths between 337 and 620 nm generated by a 337-nm nitrogen laser that pumped ten different dye cuvettes on a rapidly rotating wheel. The result was a fast EEM of the tissue collected *in vivo*. By coupling white light (~350–700 nm) into the same delivery fiber placed in contact with the tissue surface, diffuse reflectance spectra from the same tissue area were also collected. These reflectance spectra corrected the measured fluorescence spectra for artifacts induced by changes in tissue scattering and absorption, rather than by tissue biochemistry, since reflectance is affected by the same scattering and absorption processes. Statistical algorithms, such as principle component analysis, were used to identify spectral differences in measured fluorescence spectra and allowed differentiation of HGD from LGD and nondysplastic Barrett's (NDB) sites. *In vivo* autofluorescence spectroscopy from this study produced a sensitivity of 100% and a specificity of 97% for distinguishing HGD vs. LGD and NDB, and a sensitivity of 79% and a specificity of 88% for distinguishing LGD and HGD vs. NDB; however, this paper did not discuss differentiation between LDG vs. HDG, and LDG and NDB.

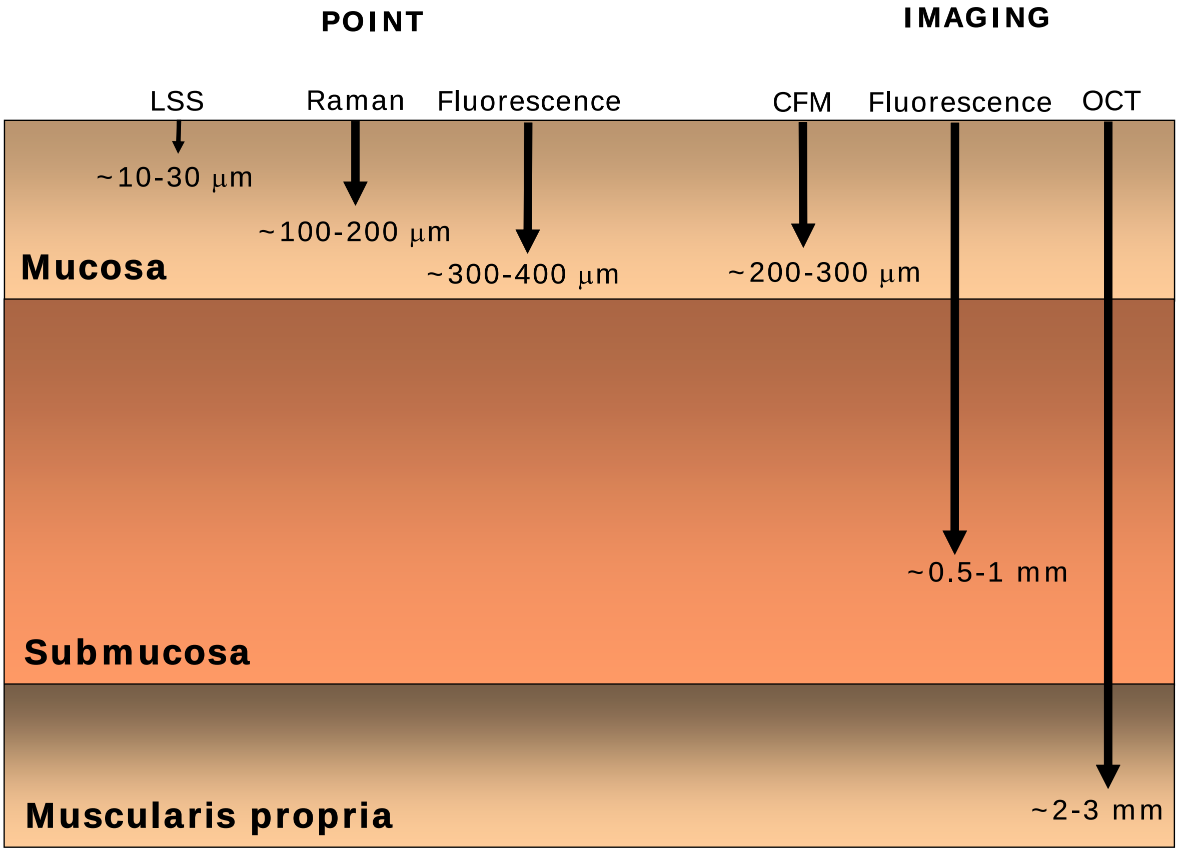

**FIGURE 2.** Relative comparison between effective tissue sampling depths of point and wide-field imaging methods.

Despite the potential benefits and relative technical simplicity of fluorescence point spectroscopy, a major drawback is the sampling of only a small volume of tissue (~1–3 $mm^3$) immediately beneath the probe tip (Fig. 2). Although capable of taking multiple readings quickly, this technique is dependent on placing the probe in the right spot (as for biopsy). Targeted sampling is currently only possible with lesions that are visible. This inherently limits the sensitivity. In addition, unlike imaging, the spectral information lacks contextual information, which is often needed to distinguish abnormal from surrounding normal tissues.

Instruments that can produce fluorescence images of high resolution and in real time, to complement WLE, have recently become available, enabling *in vivo* imaging to interrogate large areas of the mucosal surface in parallel with WLE examinations. WLE images are generated from primarily diffuse reflectance (with some contribution from specular reflectance), when the different wavelength components of a broadband (white) light source are multiply scattered and absorbed in tissue. In WLE, the illuminating and reflected light photons have the same wavelength, in contrast to fluorescence where the emitted fluorescence photons have longer wavelengths than the (near) monochromatic excitation light. Since the light scattering and, particularly, absorption in the tissue are wavelength dependent, the different wavelengths effectively interrogate the tissue to different depths (red being the most and blue the least penetrating). The resultant image provides the endoscopist with visual clues to the tissue surface topography and underlying patterns of vasculature.

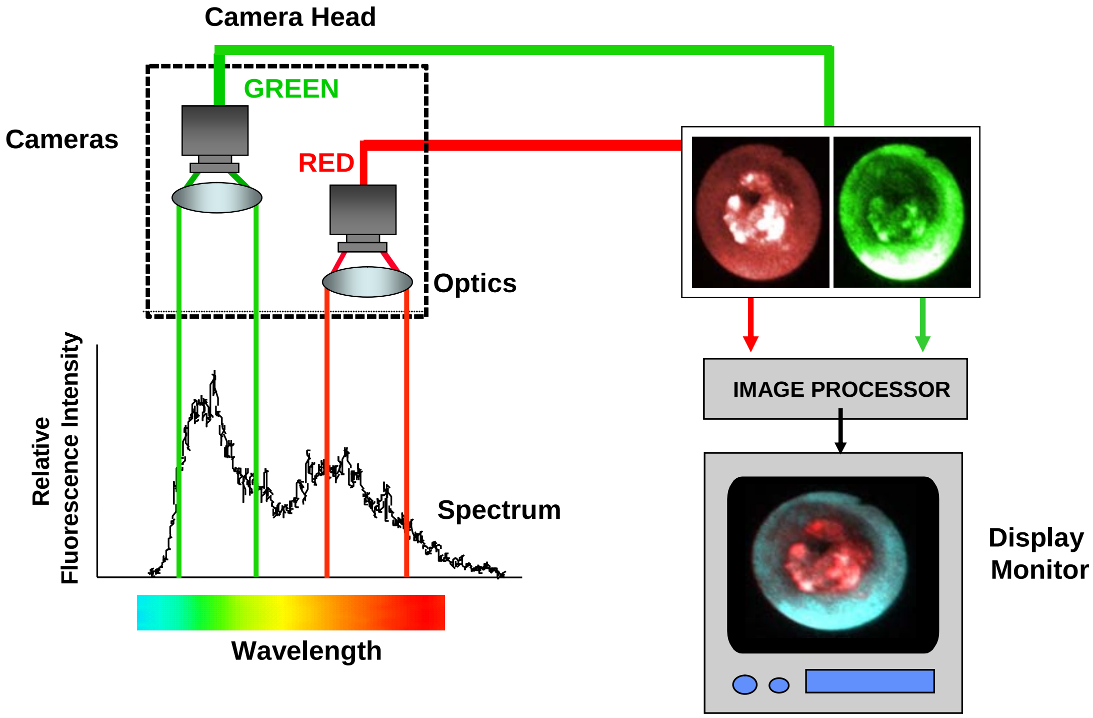

**FIGURE 3.** Schematic diagram illustrating the individual red and green fluorescence (reflectance) channels that make up the final displayed fluorescence pseudocolored image.

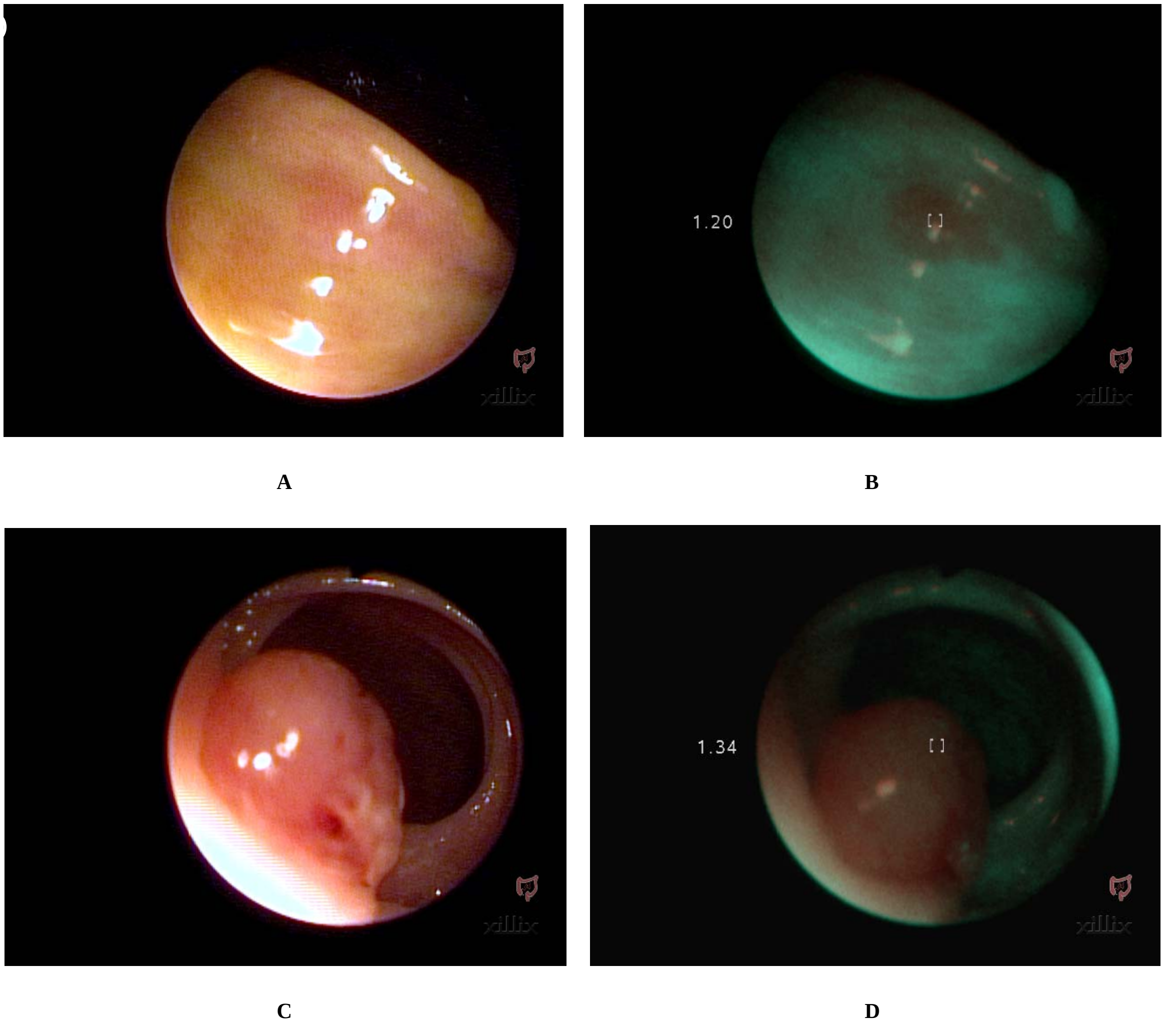

**FIGURE 4.** Typical example of (A) WLE image of a diminutive colonic adenoma and (B) the corresponding light-induced fluorescence image (pseudocolored) taken with the LIFE-II imaging system (Xillix Technologies Corp.). (C) The white light and (D) corresponding fluorescence image of a large colonic adenoma. Note the red fluorescence of both lesions compared with the cyan-colored normal mucosal background in both images.

Fluorescent images are formed using only selected emission wavelength bands. There are various ways in which this can be achieved. For example, the emission bands can be selected using special optical filters, and then detected by separate cameras to form the final displayed fluorescence image (Fig. 3). Thereby, real-time, false-color fluorescence images of the tissue can be viewed, switching rapidly between this and WLE. Such a system was first demonstrated for the bronchus as a screening tool for dysplasia and carcinoma in high-risk patients[30]. This led to a commercial system (LIFE-Lung, Xillix Technologies Corp., Richmond, BC, Canada), which used blue-light excitation, with separate red and green fluorescence imaging channels. Using LIFE in combination with WLE, the detection of moderate- to high-grade bronchial dysplasia was increased by 171%, compared with white-light bronchoscopy alone, with only a 22% decrease in specificity[31].

Currently, prototype imaging systems derived from the original lung device are being evaluated for GI endoscopy, and initial clinical feasibility studies have been reported by ourselves and other collaborating groups[32,33,34,35]. The most recent prototype uses a detachable camera module, which connects to the optical head of a conventional fiber optic endoscope. The module contains two individual high-sensitivity cameras, one for green (490–530 nm) and one for red (590–700 nm) fluorescence. The digital images are combined to produce a real-time, false-color image, where normal tissue generally appears green and abnormal tissue appears red. The ratio of red to green fluorescence is standardized over normal mucosa. The current system can be switched rapidly (~4 sec) between WLE and LIFE, allowing rapid surveying of wide areas of the mucosa. Sensitivity and specificity values are determined by correlating the positive (red fluorescence) and negative (green fluorescence) images with histologic diagnosis of corresponding biopsy samples (Fig. 4) (*also see Videos 1 and 2*).

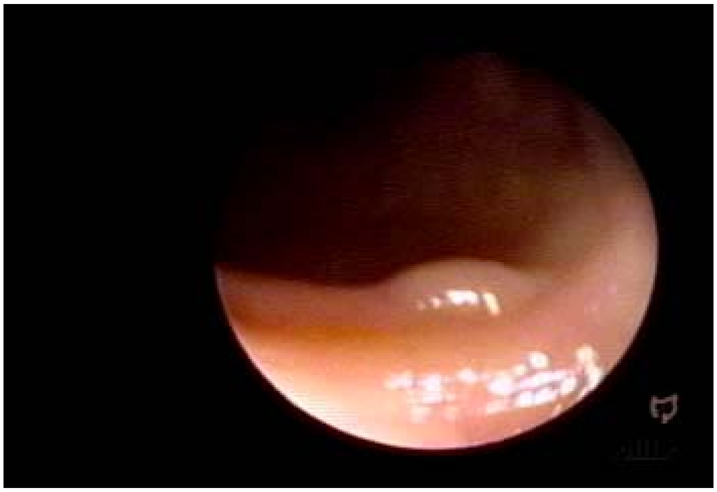

**Video 1.** Example of a hyperplastic polyp observed under white light and then with autofluorescence (Courtesy Xillix Technologies Corp.). Note that the hyperplastic polyp fluoresces similar to the normal background tissue. (**VIDEO CLIP**)

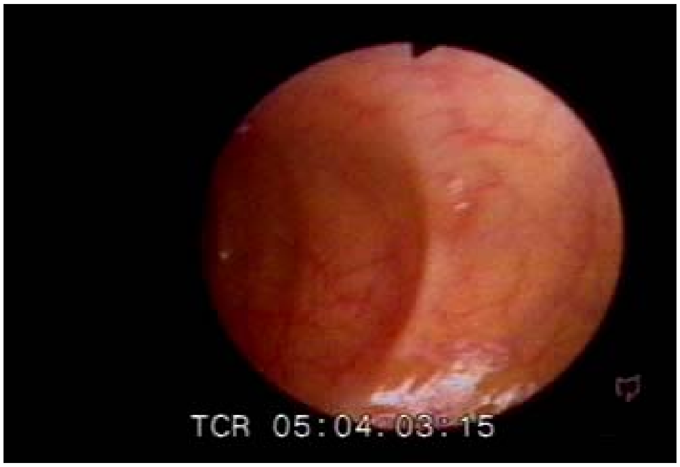

**Video 2.** Example of multiple adenomatous polyps in a patient with familial adenomatous polyposis observed under white light and then with autofluorescence (Courtesy Xillix Technologies Corp.). Note that the dysplastic polyps appear red against the cyan-colored normal background. (**VIDEO CLIP**)

In a recent prospective study by our group, we used the latest clinical prototype fluorescence imaging device called ONCO-LIFE (Xillix Technologies Corp., Richmond, BC) to examine 63 patients for whom colonoscopy was indicated for colon cancer screening or surveillance[36]. Segmental examination of the colon during withdrawal was carried out using white light (WL) followed by fluorescence light (FL) colonoscopy. Lesions missed at WL examination were recorded as false-negatives. White light colonoscopy was able to detect 101 positive lesions and 18 additional adenomas were identified only at FL examination. The addition of FL imaging resulted in an increased adenoma detection rate of 17.8% (18/101), which included diminutive and flat adenomatous lesions. The accuracy of endoscopic diagnosis for dysplastic lesions improved from 58 to 72%, and for hyperplastic lesions from 62 to 73% with the use of WL+FL. The sensitivity (detection and correct endoscopic diagnosis) using WL+FL was 80.7% compared to 58.0% with WL only, yielding a relative sensitivity of 1.39. In the noninflammed colon, we had no false-negatives. False-positive lesions have been seen with acute colitis, both infective and idiopathic. Diminutive polyps are extremely common and, because the endoscopist cannot usually determine whether these are hyperplastic or adenomatous, are removed or fulgurated. This can be labor intensive. The ONCO-LIFE imaging system was able to differentiate between hyperplastic polyps, which have no known malignant potential, and adenomatous polyps, which should be removed. Detection of lesions of this type is the ultimate goal of any effective screening program and could have a significant impact on survival.

Villous adenomas are often sessile and can cover several square centimeters of colonic mucosa. Their removal usually involves the submucosal injection of saline prior to polypectomy. Complete removal is sometimes problematic and recurrence is sometimes difficult to identify with WLE in the area of the scar. Fluorescence endoscopy, however, facilitates this nicely.

Although cancer of the esophagus in most Western countries is relatively uncommon in comparison with cancer of the colon, the 5-year survival rate is only 5%, primarily because most patients at the time of diagnosis have advanced disease and, therefore, reduced survival despite aggressive surgical and radiotherapeutic intervention. Considerable interest is being focused on Barrett’s and the rising incidence of adenocarcinoma in the distal esophagus. The main selection factors in identifying patients with Barrett’s are symptoms of chronic reflux, anemia, a possible family history of Barrett’s, and adenocarcinoma. Patients known to have Barrett’s are usually enrolled in a surveillance program. The endoscopic recognition in Barrett’s esophagus of nodules, discrete ulceration, and strictures draws the endoscopist to biopsy these obvious lesions. Most patients with Barrett’s esophagus will not have recognizable lesions and the usual approach is a systematic biopsy protocol (i.e., Seattle), which traditionally recommends four-quadrant biopsies at least every 2 cm. It would be useful to have an optical technology that would better identify dysplastic areas, resulting in better mapping and a reduced number of biopsies. Such a technology is not currently available. However, there has been considerable effort in such a technology. First, there is a method of wide scanning that functions as a “red flag” or “waving hand” to draw the attention of the endoscopist to an area of suspicion for diagnosis and therapy (i.e., mucosal resection). The methodology includes the use of chromoendoscopy, which has been available over the last 10 years, and the more recently described autofluorescence and narrow band imaging (NBI) techniques. The earliest implementation of tissue autofluorescence for identifying dysplasia in Barrett’s was based on point spectroscopic measurements using fiber optic probes placed in gentle contact with the mucosal surface at multiple sites[25]. Results from these earliest spectroscopic studies formed the foundation of today’s autofluorescence imaging systems. The main advantage of current fluorescence endoscopic imaging systems is the capability of scanning wide mucosal areas. Early endoscopic prototypes using fiber optic technology were disappointing because of a high incidence of false-positives, and this continues to plague even the current video-based prototypes. The explanation for this high incidence of false-positives is thought to be related to background inflammation[37].

There is a paucity of published studies using the latest generation of video-based autofluorescence endoscopes in the detection of dysplasia in Barrett’s. Results from the Amsterdam group showed that autofluorescence (AFI; Olympus Optical Co. Ltd., Tokyo, Japan) compared with WLE led to a 40% false-positive rate in an uncontrolled study of a highly selected group of patients[38]. This study highlights the fact that even with the latest prototype, the reduction of false-positives requires a second optical technology (i.e., NBI). In the Amsterdam study, this was the use of a prototype NBI system (Olympus) with a high magnification capability. Although the results of this proof-of-principle study were promising, the fact that two different endoscopes had to be used sequentially will not likely be acceptable in clinical practice. It does, however, support the concept of a multiplatform endoscopic system in the future that incorporates high-resolution autofluorescence and NBI with magnification. Whether this will be technically and economically feasible will remain a challenge for industry and the marketplace. It is noteworthy that results of a similar application combining both autofluorescence (e.g., AFI) and NBI for the detection of diminutive colonic polyps and dysplasia in inflammatory bowel disease have not been published.

## CLINICAL EXPERIENCE OF EXOGENOUS FLUORESCENT DRUGS

To date, several key reports have been published on the clinical effectiveness of prodrugs, such as ALA, to detect preneoplastic GI lesions. Messmann et al. reported an experimental ulcerative colitis (UC) rat model in which ALA-PpIX fluorescence (UV excitation; i.v. administration of ALA at 25–200 mg/kg body weight; visualization 1–4 h later) was used to enhance the detection of LGD lesions[39]. The best results were obtained with 75 mg/kg ALA, for which dysplasia (LGD and HGD) was detected with a sensitivity of 92%, although the specificity was disappointing at 35%. Focal lesions as small as five abnormal crypts were observed in *ex vivo* tissue samples, which is a significant finding, since aberrant crypts are thought to be the earliest precursors to dysplastic lesions in the colon[40,41]. The major limitation of this study was that UC also showed appreciable concentrations of PpIX, resulting in high false-positive rates, although identification of LGD against an active colitis background may be difficult even for the skilled pathologist using biopsied tissues[42]. Lowering the ALA dose increased the specificity, with a notable decrease in the false-positive rate, although at the price of reduced sensitivity, which may have been due, in part, to the use of the unaided eye for detecting the fluorescence. Messmann et al. suggested improving the technique by topical administration of the ALA, by optimizing the time period between drug administration and fluorescence observation, and by attempting to detect HGD in quiescent colitis. An alternative may be to keep the ALA dose low and increase the detection sensitivity; for example, using a high-sensitivity imaging system (see below)[19].

Another recent article by Messmann et al. reports six patients with dysplastic lesions in the GI tract, two of whom had Barrett's, one had LGD, and the other had HGD[43]. The patients drank 10–20 mg/kg ALA and the endoscopic examination was performed 1–6 h later using blue light excitation and compared with conventional WLE. In this limited and selected group of patients, Messmann et al. claimed that low-dose ALA facilitated the identification/differentiation of dysplastic lesions in a background of Barrett's, and showed histological proof that the red fluorescent area was dysplastic. Surprisingly, an area of adjacent ulceration did not fluoresce red, despite the fact that inflammation is a confounding feature that is thought to be responsible for high false-positive rates. In this study, a 40% false-positive biopsy rate was attributed to inflammation. Low-dose oral ALA resulted in higher background red fluorescence in normal duodenal and squamous epithelia compared with normal stomach mucosa and Barrett's epithelium. However, this is contradicted by recent findings of van den Boogert et al. in a rat model of Barrett's using intravenous ALA, who concluded that there was no selectivity in ALA-induced fluorescence between normal squamous and adjacent Barrett's mucosa[44].

In a recent *in vivo* study, Ortner et al. demonstrated in 53 Barrett's esophagus patients that it was possible to differentiate LGD from nondysplastic Barrett's mucosa during endoscopy based on time-delayed laser-induced fluorescence endoscopy of ALA-induced PpIX in dysplasia[45]. ALA was sprayed on the mucosa and about 1–2 h later, biopsy specimens were collected based on point spectroscopy measurements of the time-delayed fluorescence intensity ratios of PpIX. Despite taking fewer specimens, dysplasia was detected at a rate 2.8-fold higher compared with screening endoscopy. In addition, three early cancers were detected for the first time. In preliminary quantitative *in vivo* studies, Brand et al.

administered 10 mg/kg of ALA orally 3 h to 20 Barrett's esophagus patients prior to endoscopy[46]. Using 400-nm excitation, PpIX fluorescence intensity at 635 nm was detected spectroscopically and compared with histopathologic diagnosis of biopsies taken immediately after the fluorescence measurements. By using PpIX fluorescence alone, HGD was distinguished from nondysplastic tissue types with 77% sensitivity and 71% specificity. Decreased autofluorescence was particularly found in nodular HGD. By using the fluorescence intensity ratio of 635 nm/480 nm, nodular HGD could be differentiated from nondysplastic tissue with 100% sensitivity and 100% specificity. Despite these notable findings, larger clinical trials are awaited to confirm the diagnostic efficacy of ALA for Barrett's esophagus.

Studies are also currently under way at our institution to assess the combined role of ALA in diagnostic fluorescence imaging (and ablation using PDT) in Barrett's esophagus (Fig. 5). Interestingly, preliminary data on LIFE imaging using very low-dose oral ALA (i.e., ~2 mg/kg) have shown selectivity of ALA in Barrett's mucosa relative to squamous mucosa that was not observed at higher doses. In our study, it was confirmed that there does not seem to be a combination of ALA dose or time interval between (systemic) administration and imaging that gives adequate reliability. The problem again is that there is high, and highly variable, PpIX synthesis in the nondysplastic Barrett's esophagus itself that gives a confounding fluorescence background[47].

ALA-PpIX fluorescence has also been studied in a variety of other organs, such as the bladder[48], oral cavity[49], and brain[50]. Peng et al. recently reviewed the use of ALA for PDT[13], while Marcus et al. summarized both the clinical and preclinical development of ALA as fluorescence diagnostic agent[51]. Further studies will continue to investigate the complementary aspects of exogenous fluorescent compounds and fluorescent imaging systems with conventional endoscopy. Their use in everyday endoscopic practice will be influenced by extra costs, regulatory approval, possible drug-related toxicities, and overall cost effectiveness as part of a screening program.

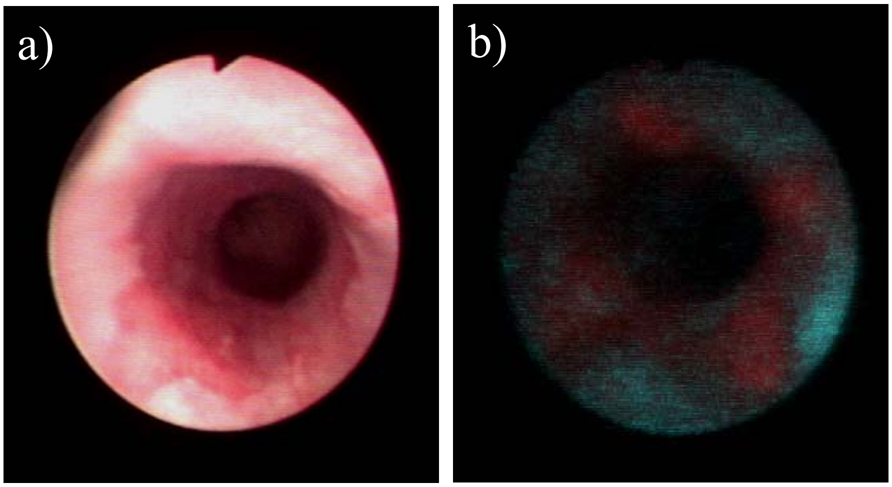

**FIGURE 5**. *In vivo* example of (a) WLE image of Barrett's esophagus and (b) corresponding fluorescence image showing red ALA-induced PpIX fluorescence selectively localizing on Barrett's mucosa relative to normal squamous mucosa, which appears cyan colored (ALA dose 2 mg/kg, image taken at 1 h postadministration).

## RAMAN SPECTROSCOPY

In tissue, most light photons are scattered without a change in their energy or wavelength (elastic scattering that is responsible for diffuse reflectance in WLE). However, the Raman effect is an inelastic scattering process, where the scattered photon's energy is changed and this Raman scattered light is shifted to a lower frequency, i.e., longer wavelength. The molecular information contained in the Raman emission spectrum can be extracted by using a spectral analyzer, yielding a "fingerprint-like" signature for the tissue sample. Since molecular bonds possess a unique pattern of Raman spectral peaks, the molecular composition of a tissue sample can be determined.

Raman spectroscopy continues to be evaluated as a means of obtaining detailed information about the biochemical composition of tissue, since it is sensitive to a wide range of specific biomolecules, such as proteins, lipids, and nucleic acids. Since the onset of cancer is accompanied by changes in biochemical composition, Raman scattering offers a potentially powerful diagnostic technique.

Unlike fluorescence and reflectance spectra, Raman spectra are "molecule specific" and offer much narrower spectral features from signals obtained as deep as 500 μm from the tissue surface. However, since the Raman effect comprises a very small fraction (about 1 in 107) of the incident photons, the Raman effect is more difficult to implement than fluorescence. Raman signals are much weaker than autofluorescence and they can be masked by the broad-band fluorescence background. In addition, specially designed optical fiber probes are required to minimize the fluorescence and Raman signals generated in the probe itself.

Generally, *in vivo* Raman spectroscopy has used near infrared (NIR) light (~700–1300 nm) to produce Raman spectra, since it both penetrates deeply into tissue and minimizes tissue autofluorescence compared with visible light excitation. UV light can also be used to produce tissue Raman spectra, with the advantage of producing tissue autofluorescence that is well separated from the detected Raman spectral bands; and special optical filters can reduce unwanted background fluorescence. Also, in UV resonance Raman (UVRR) spectroscopy, certain Raman spectral bands can be appreciably amplified using excitation light corresponding to a particular absorption band. However, compared with NIR, UV light does not penetrate deeply into tissues and is potentially mutagenic, thereby limiting its use clinically.

Some of the first results using Raman spectroscopy on *ex vivo* GI tissue samples were reported by Shim et al.[52]. In this study, potential artifacts in Raman spectroscopy due to a variety of handling procedures of *ex vivo* tissue biopsy samples were determined; and using a specially designed optical probe, *ex vivo* spectra were able to differentiate dysplasia from intestinalized dysplasia in esophageal biopsies with a sensitivity of 77% and a specificity of 93%.

## CLINICAL EXPERIENCE OF RAMAN SPECTROSCOPY

Several investigators have used different configurations to acquire *in vivo* Raman spectra, but there are few reports for the GI tract[93]. Recently, Shim et al. designed and built a fiber optic NIR device for *in vivo* Raman spectroscopy (IVRS) measurements, and reported the first *in vivo* Raman spectra of human GI tissues measured during routine clinical endoscopy with acceptable signal-to-noise ratio (SNR) and short collection times[53]. This was achieved by using the system with an optically filtered fiber optic probe (~2 mm diameter) that was passed through the endoscope instrument channel and placed in contact with the tissue surface. Background signals were suppressed and the light collection efficiency was optimized. Spectra were obtained with good SNR in ~5 sec. The effects on the spectra of varying the pressure of the probe tip on the tissue and of the probe-tissue angle were insignificant.

The spectra from normal and diseased tissues revealed only subtle differences. For example, initial *ex vivo* studies on Barrett's esophagus samples (207 nondysplastic, 53 dysplastic) demonstrated slight differences in spectral line shapes in the 1100–1800 $cm^{-1}$ range, but no specific prominent changes in peak intensity or position. Sophisticated computational techniques, such as principle component analysis and artificial neural networks, are being investigated, which have the advantage that they analyze the full Raman spectrum and, hence, the full diagnostic content in order to distinguish subtle spectral differences among these spectra. One type of neural network analysis differentiated esophageal dysplasia from metaplasia with a sensitivity of 77% and a specificity of 93%. These algorithms are being refined to improve on these results and to give the best discriminating power in classifying the various dysplastic grades (indefinite vs. LGD vs. HGD)[54,55]. Once optimized, these algorithms can be used in a prospective fashion to assess the potential of Raman spectroscopy for Barrett's tissue differentiation. Moreover, the recent first demonstration of the feasibility of obtaining *in vivo* Raman spectra of adenomatous and hyperplastic colonic polyps with NIR excitation is a critical step in initiating systematic clinical trials to determine the diagnostic accuracy of Raman spectroscopy in Barrett's esophagus[56].

Although Raman microscopic imaging can be done *ex vivo*, the weakness of the signals may never allow real-time endoscopic *in vivo* Raman imaging unless a signal enhancement method, such as the use of metal nanoparticles, is made (surface enhanced Raman)[57]. However, an important advance in understanding the biochemical basis for the observed spectral changes has been recently reported from a U.K. group using microscopy to map the full Raman spectrum in *ex vivo* tissue specimens[58]. By fitting the spectra to basis spectra of known components, the relative concentration of each component was determined for each image pixel, thus generating a composite "intensity" map. Signatures for DNA, oleic acid, collagen I, and actin were associated with HGD compared to normal squamous esophagus, in which glycogen showed significant peaks. Further studies on a larger number of samples, including the full assortment of esophageal pathologies, are needed, but this study has shown the value of such detailed spectral mapping. It would, of course, be a significant advance if such maps could be produced *in vivo* during endoscopy, since this could further improve the diagnostic power of the Raman characteristics.

## ELASTIC LIGHT SCATTERING SPECTROSCOPY

Light scattering spectroscopy (LSS) is based on white light reflectance in which photons incident on tissue are back scattered without a change in their wavelength, and provides structural information about tissue *in situ*. LSS measurements are performed with visible light (400–700 nm) using fiber optic probes placed at the tissue surface through the endoscope and detect the relative intensity of back-scattered photons. These measurements have been shown to be sensitive to both endogenous tissue scatterers (i.e., cell nuclei and mitochondria) and tissue absorbers (i.e., hemoglobin). Since the optical probe detects scattered photons from as deep as the submucosa, information about the mucosal layer must be obtained by separating the contribution of multiply scattered light from deep in the tissue. Methods to do this include spectral subtraction or the use of cross-polarization. In this way, singly scattered photons originating primarily from the mucosa can be obtained. Tissue transformation is associated with changes in the density and/or size of endogenous scatterers within the mucosal layer. These can be measured from the fine structure in the spectra and correlated with histopathology. One of the hallmarks of dysplastic change is that nuclei enlarge and become crowded, and LSS is able to measure the size distributions of epithelial cell nuclei. In this way, direct quantitative measurement of nuclear enlargement, crowding, hyperchromaticity, and amount of chromatin is provided, which aids in clinical diagnosis. LSS is a noninvasive method that does not require expensive laser light sources, but instead is performed with white light that yields a strong signal in real time (<1 sec for a spectroscopic reading)[59,60,61]. Despite the advantages of requiring a simplified and less expensive spectroscopic detection system, and similar to point fluorescence spectroscopy, the main disadvantage is that the LSS optical probe has a limited sampling volume. However, while technically difficult, LSS is currently being evaluated for *in vivo* imaging. Gurjar et al. have extended this technique to imaging using a CCD camera and a set of narrow band filters to sample the reflectance spectrum and subsequently extracting the nuclear size parameters at each image pixel point[62]. However, this has not been reported to date applied to Barrett's esophagus. There are major technical challenges to achieve adequate spatial resolution and speed to make this a true endoscopic imaging technique that can be implemented at reasonable cost. Detailed studies will be needed to determine what spatial and spectral resolutions are needed to distinguish dysplasia in Barrett's esophagus.

## CLINICAL EXPERIENCE OF LIGHT ELASTIC SCATTERING SPECTROSCOPY

The potential of using LSS *in vivo* to detect epithelial nuclear crowding and enlargement in Barrett's esophagus was evaluated by Wallace et al.[60]. Diffusely reflected white light was spectrally analyzed to obtain the size distribution of cell nuclei in the mucosal layer, from which the percentage of enlarged nuclei and the degree of crowding were determined. Dysplasia was assigned if more than 30% of the nuclei exceeded ~10 μm in diameter, and the histologic findings were compared with those of four pathologists blinded to the light-scattering assessment. Using this threshold, the sensitivity and specificity for detecting dysplasia (LGD and HGD) were both 90%.

Georgakoudi et al. assessed the potential of three spectroscopic techniques (fluorescence, reflectance, and LSS), individually and in combination, for evaluating LGD and HGD in 16 patients with Barrett's esophagus[29]. Fluorescence spectra at 11 excitation wavelengths (337–610 nm) and a reflectance spectrum (350–700 nm) were collected in about 1 sec from each tissue site by an optical fiber probe before biopsy. The measured fluorescence spectra were combined with the reflectance spectra to extract the intrinsic tissue fluorescence. The reflectance spectra provided morphologic information about the bulk tissue, whereas LSS was used to determine cell nuclear crowding and enlargement in the Barrett's epithelium. Significant differences were observed between dysplastic and nondysplastic Barrett''s esophagus in terms of intrinsic fluorescence, bulk scattering properties, and levels of epithelial cell nuclear crowding and enlargement. The combination of all three techniques (called Tri-Modal Spectroscopy) resulted in high sensitivity and specificity for separating high-grade from non-high-grade (sensitivity of 100%, specificity of 100%, respectively) and dysplastic from nondysplastic epithelium (sensitivity of 93%, specificity of 100%, respectively). This study concluded that intrinsic fluorescence, reflectance, and light-scattering spectroscopies provide complementary information about biochemical and morphologic changes that occur during the development of dysplasia in Barrett's esophagus. Further studies to test the capabilities to differentiate between various grades of dysplasia are awaited.

Lovat et al. assessed the potential for elastic LSS to detect HGD or cancer within Barrett's esophagus in 81 patients[63]. Measurements collected *in vivo* were matched with histological specimens taken from identical sites within the Barrett's esophagus field, and biopsies were classified as either "low risk" (nondysplastic or LGD) or "high risk" (HGD or cancer). Elastic scattering spectroscopy detected high-risk sites with 92% sensitivity and 60% specificity, and differentiated high-risk sites from inflammation with a sensitivity and specificity of 79%. This group concluded that if LSS was used to target biopsies during endoscopy, the number of low-risk biopsies taken would decrease by about 60% with minimal loss of accuracy. Furthermore, a negative spectroscopic result would exclude HGD or cancer with an accuracy of >99.5%. The authors suggest that LSS has the potential to target conventional biopsies in Barrett's surveillance, which would save significant endoscopist and pathologist time and increase the cost effectiveness. However, this technique now requires validation in prospective large studies[63].

To date, the potential of using LSS *in vivo* to detect epithelial nuclear crowding and enlargement in human colon has been reported in one clinical study by Dhar et al.[64]. The capability of detecting changes in nuclear size and density could potentially be useful in identifying dysplastic lesions in the colon, despite the limited sampling volume. Dhar et al.[64] used the elastic light scattering spectra obtained from 138 sites in 45 patients at colonoscopy. These spectra were then compared with conventional biopsy specimens taken from the same site, including normal colonic mucosa, hyperplastic polyps, adenomatous polyps, chronic colitis, and colon cancer. Spectral analysis was carried out with a validated computerized model that used principal component analysis followed by linear discriminant analysis. The sensitivity and the specificity of differentiating adenomas from hyperplastic polyps was 84 and 84%, respectively; for cancer from adenomatous polyps, 80 and 75%, respectively; for colitis from normal tissue, 77 and 82%, respectively; and for dysplastic mucosa (from polyps) from colitis, 85 and 88%, respectively. Thus, it was concluded that the ability to differentiate colonic lesions accurately makes ESS a potentially useful tool to make real-time diagnosis during colonoscopy by facilitating targeting biopsies in dysplasia surveillance in inflammatory bowel disease[64].

## IMMUNOPHOTODIAGNOSTIC ENDOSCOPY

For the past 20 years, radiopharmacology has relied on the highly specific reactivity of the antigen-antibody complex. For example, radiotherapeutic agents are commonly conjugated to monoclonal antibodies directed against tumor-related or tumor-associated antigens. These are used to target tumor cells selectively for destruction based on the inherent overexpression of a particular tumor-associated antigen relative to normal tissues[65]. Adapting this principle for fluorescence endoscopy involves the conjugation of a fluorophore dye to a monoclonal antibody or other tumor-targeting moiety, thereby producing a "fluorescent contrast agent", which may enhance the fluorescence contrast between tumors and the surrounding normal tissue during *in vivo* fluorescence endoscopic imaging. Typically, these dyes are excited in the red range (>600–700 nm) and emit NIR fluorescence efficiently. They have adequate stability for labeling *in vivo* and produce fluorescence that is detectable through millimeter thicknesses of tissues[66]. Recent improvements in monoclonal antibodies and their derivatives (i.e., fragments), the development and commercial availability of NIR-emitting fluorophores, and the availability of high-sensitivity digital cameras in this spectral region have made tumor-localization using fluorescence contrast agents practical and attractive. Optimal fluorescent dyes can be selected based on their photophysical and spectral properties independent of their tumor-localizing properties[67].

Recent animal studies have demonstrated that fluorophore labeling of monoclonal antibodies produces adequate sensitivity and improved tumor-to-normal contrast[68,69]. Further studies continue to investigate ways to improve the binding characteristics of such fluorescently labeled contrast agents *in vivo*[70]. However, many difficulties remain with this approach. For example, until recently, most monoclonal antibodies were raised in nonhuman hosts (i.e., mice) resulting in a host-immune response against them when used in patients. This not only causes the antibodies to be eliminated quickly, but also forms immune complexes that cause damage to the kidneys[71]. However, "humanized" monoclonal antibodies have become available recently. In addition, whole antibodies bound in human tumors do not exceed $10^{-5}$ of the administered dose per gram of tumor; hence requiring large amounts of injected conjugated monoclonal antibody, long exposure times, and high sensitivity to achieve adequate tumor brightness and contrast. This limitation is due to the pharmacokinetic properties of conjugated whole antibodies. The production of antibody fragments, smaller than the whole antibody, has resulted in some improvements in pharmacokinetics and tissue labeling. The use of NIR-conjugated smaller antibody fragments was demonstrated to produce rapid tumor uptake, better penetration (at the expense of reduced circulation time), more homogeneous tumor penetration, and reduced immunogenicity[72,73].

Fluorescent dyes can also be targeted to tumor tissues by means other than monoclonal antibodies, such as fluorescently labeled enzyme-sensing probes[74,75]. These groups demonstrated that specific enzyme activity in a tumor could be imaged by fluorescence contrast agents *in vivo* and that even microscopic lesions (~50 μm in diameter), undetected by white light imaging, were readily detected by fluorescence.

Compared to organic fluorophores, fluorescent quantum dot (QDot) semiconductor nanoparticles have high brightness, broad excitation spectra, and relatively narrow and size-dependent (and, hence, selectable) fluorescence emission spectra, and low photobleaching. This gives QDots several potential advantages for their use in fluorescence endoscopy, some of which could increase the efficacy of fluorescence-based diagnostics, including Barrett's esophagus endoscopy. Currently, recent exploratory research in our laboratory is assessing the use of QDot-based fluorescence contrast agents for endoscopic imaging. Examples of this work include the evaluation of QDot agents *in vitro* in established GI tumor cell lines (Fig. 6), and *in vivo* in relevant animal models of colonic adenomas (Fig. 7) and Barrett's esophagus (Fig. 8).

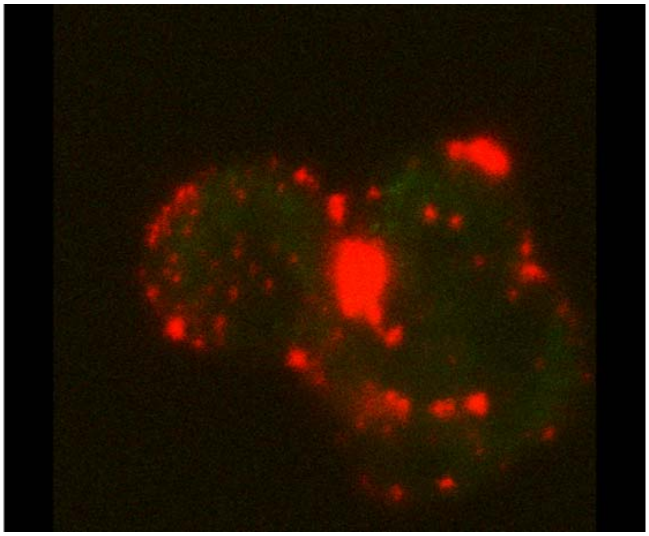

**FIGURE 6**. Example of the *in vitro* use of fluorescent QDots conjugated to detect the cell-surface receptor (tumor-associated antigen-72) overexpressed in LS174T human adenocarcinoma cells. Bright red fluorescence from the QDot probes is observed on the cell surface of the colon tumor cells (which also emit a weak green autofluorescence), indicating specific targeting (458-nm excitation; green channel = 500–530 nm; red channel = 650–710 nm).

Overall, the long-term goal for tumor-targeting fluorescence contrast agents is to translate their use from preclinical animal cancer models to the clinical environment. Currently, regulatory restraints prevent/restrict the testing of such agents in humans without careful studies confirming that such exogenous diagnostic agents are effective, nontoxic, and safe. Albeit toxicology studies are still awaited, the basic methodology of using such molecularly-specific contrast agents continues to be investigated at the preclinical level.

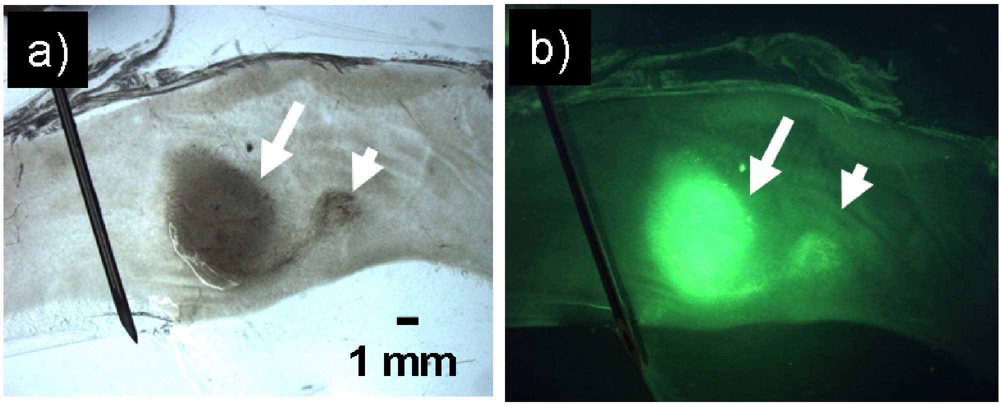

**FIGURE 7.** Example of *in vivo* targeting of carcinoembrionic antigen (CEA) in colonic adenomas developing in a mouse model. (a) Transmitted white light image of resected mouse colon showing dysplastic lesions (arrows) and (b) corresponding fluorescence image of the bright green–emitting QDots conjugated to a monoclonal antibody recognizing CEA, which is overexpressed in dysplastic lesions. Note the intense green fluorescence of the adenomas, indicating selective targeting of the agent and significant enhancement of the tumor-to-normal contrast.

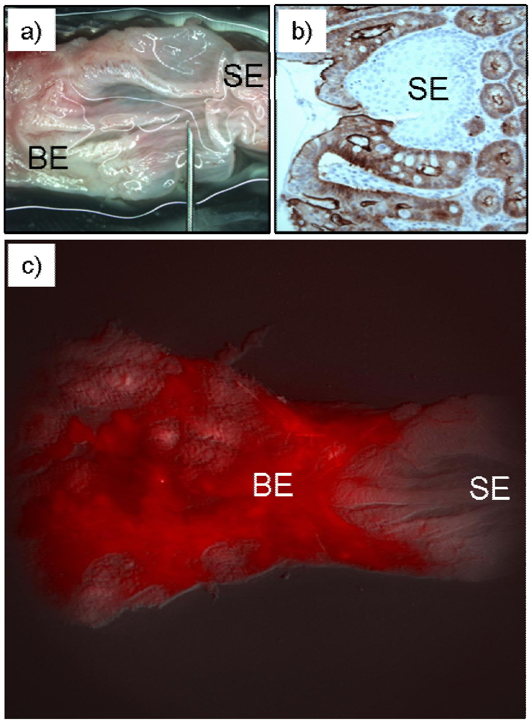

**FIGURE 8.** Example of *in vivo* targeted labeling of QDots in Barrett's esophagus rat model. (a) White light image of resected rat esophagus, (b) *ex vivo* tissue section stained immunohistochemically for villin (brush-border protein) expression, and (c) fluorescence image of the luminal surface of the esophagus, with topical application of a conjugate comprising 660 nm–emitting QDots conjugated to an antibody targeted to the brush-border protein called villin that is overexpressed in Barrett's esophagus tissue (BE = Barrett's mucosa, SE = squamous esophageal mucosa).

It is envisaged that once optimized and validated in prospective clinical trials, these modalities are likely to be used by the endoscopist as guiding tools for targeted biopsies, and to assess lesion margins and adequacy of endoscopic treatment more accurately. It is conceivable that optimal detection will require a combination of techniques (multimodal diagnosis). For example, a suspicious area could be identified initially by a wide-field imaging modality, such as fluorescence endoscopy, and then further characterized (or graded) by spectroscopy or by a high-resolution technique, such as confocal fluorescence microendoscopy. Furthermore, such optical probes may not only detect superficially localized lesions, but also determine the extent of local tumor infiltration and local regional metastases (e.g., infiltrated lymph nodes). Ultimately, diagnostic performance, ease of use, and cost effectiveness will determine whether these modalities become part of standard endoscopic practice. It is anticipated that such fluorescence-based molecular endoscopic imaging will ultimately improve early diagnosis, staging, and stratification of patients with GI cancers.

We are entering a new age in diagnostic endoscopy, one in which optical imaging will permit detection of early preneoplastic disease at the "*molecular level*". We anticipate that the next evolutionary stage for fluorescence-based endoscopic imaging will be the concurrent improvement of existing fluorescence endoscopic imaging technologies coupled with the development of "multiplexed" fluorescent contrast agents that simultaneously and specifically target tumor-associated biomarkers. These agents may be delivered in "cocktail" form and applied either topically or systemically, thus facilitating real-time *in vivo* detection of very early preneoplastic lesions, targeted biopsies and guided surgical resection, detection of residual disease or recurrence, and aid in detection of metastatic disease, for examples. We acknowledge, however, that before such hopeful results can be realized or even brought to the clinic, there are a number of important research areas that require future work, including assessment of target specificity, optimal dosage and time intervals, pharmacokinetics and tissue microdistribution, and toxicity must be evaluated. Furthermore, the use of alternate targeting moieties, such as GI tumor-targeting peptides[76], antibody fragments[77], and more exotic agents, such as molecular beacons[75], must be considered. Such studies will be further complemented by the implementation of hyperspectral imaging in fluorescence endoscopy[78], especially for multiplexed imaging of tumor-targeting fluorescent cocktails.

The newly emerging fields of fluorescence endoscopy, tumor biomarker discovery, and nanotechnology synergistically combine to create a new era of "molecular endoscopy" that offers great hope for detection of dysplasia and early cancers in the GI tract. Together with promising autofluorescence-based imaging devices, it is envisioned that a panel of tumor biomarkers labeled individually with bright fluorescent probes could potentially be used to detect the very earliest presence of cancer and further stratify patients for risk of developing cancers. In the next few years, the combination of these tumor contrast-enhancing strategies with novel optical imaging techniques will significantly advance our attempts to improve diagnostic endoscopic imaging and bring it closer to the "*molecular level*" of disease detection currently offered by the modern molecular pathology laboratory.

## CLINICAL EXPERIENCE OF IMMUNOPHOTODIAGNOSTIC ENDOSCOPY

Preliminary *in vivo* evaluation of fluorescence contrast agents in patients has been reported in a limited number of studies. Early vascular changes were assessed in Crohn's disease in a prospective fluorescence endoscopic study of ten asymptomatic patients using unconjugated 10% sodium fluorescein[79]. Muguruma et al. used indocyanine green (ICG)–labeled antibody against epithelial membrane antigen (EMA) and an infrared flexible endoscopy to study *ex vivo* human tissues[80]. This demonstrated fluorescent antibody staining of *ex vivo* normal human esophageal tissues, but did not evaluate diseased tissues. In another study with *ex vivo* paraffinized tissues of human esophageal, gastric cancer, and colorectal cancer, Bando et al. developed NIR-excited ICG-sulfo-OSu, conjugated to antisulfomucin and anti-MUC1 antibodies to show that antibody staining patterns varied depending on the organs, the histological types, and depth of the cancers[81]. Although these authors noted the difficulty of adapting this staining method *in vivo*, where the antibody agent would be administered to the luminal surface, because of such problems as surface mucus and pH, Hayashi et al. suggested that vital immunohistochemical staining of ICG-conjugated anti-EMA antibodies on nonfixed freshly excised tissue samples is possible under optimized conditions[82]. Ito et al., in a very limited number of patients, demonstrated that such immunofluorescent staining using ICG-sulfo-OSu conjugated to anti-CEA antibodies could be performed *in vivo* to detect small gastric cancers[83]. Tatsuta et al. labeled anti-CEA monoclonal antibodies with fluorescein isothiocyanate (FITC) to study *ex vivo* human gastric lesions[84]. Here, gastric mucus was removed using proteinases, sodium bicarbonate, and dimethyl-polysiloxane to improve the binding of the tumor with the labeled antibody. Although 90% of tumors showed positive fluorescence with no false-positives, and positive fluorescence was not demonstrated in benign gastric lesions, no significant relationship between positive fluorescence and tumor type or stage was found. In 1998, Keller et al. coined the term "immunoscopy" in a report on the detection of colorectal carcinomas and villous adenomas in surgically resected tissue samples with FITC-labeled anti-CEA antibody[85].

To date, only two published reports of fluorescence-conjugated monoclonal antibodies used in humans *in vivo* have appeared. The first study in a limited set of patients used intravenous administration of a monoclonal fluoresceinated anti-CEA conjugate to detect human colon carcinoma. This was based on visual observation of green fluorescence in surgically resected tumors and also *in vivo*[86]. In the second *in vivo* study of 27 patients with colonic polypoid lesions, Keller et al. used a locally administered fluorescein-labeled anti-CEA monoclonal antibody for *in vivo* fluorescence endoscopic detection of colorectal dysplasia and carcinoma[87]. Compared with fluorescence-negative normal-appearing mucosa, fluorescence was present in 19/25 carcinomas and in 3/8 adenomas; although, the technique failed in the presence of mucosal ulceration or bleeding. Larger trials to demonstrate the value of this technique for differential diagnosis are currently under way.

However, despite these encouraging initial results, several important issues must be resolved. For example, determining the optimal mode of administration of the conjugate *in vivo*, whether topical or systemic, is still unresolved. Furthermore, selection of the best tumor-associated targets (i.e., monoclonal antibodies, peptides, enzymes) is not clear, and the possibilities are seemingly endless[88,89]. Additionally, simultaneous localization of multiple reagents is made possible by labeling multiple NIR fluorophores; thus background subtraction and differential labeling of multiple tumor-associated biomarkers can be performed. Difficulties in using the fluorophore labels are mainly related to light scattering and absorption in tissues, but detection of small superficial tumors at depths of a few millimeters should be feasible with NIR fluorescence. Recent exciting developments in the development of a new class of so-called “smart nanoparticle” probes may be capable of pinpointing their *in vivo* position in diseased tissues through their magnetic properties (i.e., MR imaging), while providing information about their molecular environment with their fluorescence properties[90]. Metal nanoparticles such as those used in electron microscopy, also conjugated to a tumor-targeting agent, may provide an alternative approach, since these greatly enhance the back-scattered light intensity, thereby providing a contrast mechanism that could be used with conventional WLE[91]. Additionally, tumor-targeted fluorescence contrast agents offer the potential of reduced costs and important time delays associated with conventional multiple random biopsy protocols by providing guided tissue biopsy. Given the limitations in current fluorescence endoscopic imaging in detecting very early GI lesions or preventing false-positives due to confounding concurrent conditions (i.e., inflammation, ulceration, and bleeding), these developments significantly compliment existing fluorescence endoscopy. Certainly, prospective long-term follow-up studies will be required in order to evaluate the usefulness of such an approach.

## THE IDEAL COMBINATION OF TECHNIQUES

Several new optically based techniques are being evaluated to enhance the diagnostic capability of clinical GI endoscopy. The ideal system should function in real time and combine excellent diagnostic accuracy with wide mucosal area surveillance. A major issue is how the detection of dysplasia and intramucosal cancer will ultimately fit into the treatment algorithm: for example, who and/or what should be treated with endoscopic ablation, chemoprevention, or resective surgery? Treatment will be markedly affected by accurate staging of lesions, via super high-resolution ultrasound or optical coherence tomography (OCT). Short of replacing conventional biopsy, such technologies should provide guidance in locating optimal sites for targeted biopsy and be able to monitor ablative therapies, such as PDT. In this regard, fluorescence endoscopic imaging with its wide field of view has already detected early lesions and scars, and demonstrated reliability in differentiating hyperplastic vs. adenomatous polyps *in vivo*, and so appears most appealing and practical for screening. Additionally, fluorescence endoscopy does not require dye spraying and is relatively fast. However, many issues, such as optimal excitation and emission wavelength(s), confounding background metaplastic fluorescence (false-positives), and artifacts due to motility, remain unresolved. Additionally, it is not clear if exogenous fluorophores, such as prodrugs like ALA, will be necessary to achieve clinically useful sensitivity and specificity.

Despite its very high molecular specificity, Raman spectroscopy suffers the same weakness as all point spectroscopies in that its clinical use is limited by practicality. This is also the case for LSS, which has shown promise in differentiating dysplasia (LDG and HDG) from Barrett’s esophagus based on nuclear size and density. However, used adjunctively with imaging techniques that survey large tissue surfaces for targeting suspicious lesions, the molecular specificity of Raman spectroscopy or the sensitivity to subcellular scattering features of LSS may be useful for *in situ* diagnosis. These combinations are yet to be attempted.

In our opinion, OCT is the optimal "optical biopsy" device of the future in that it could incorporate histological-quality detail with the advantage over other endoscopic devices of providing tissue-depth information, perhaps as deep as 2000 μm compared with 250 μm as obtained with confocal fluorescence microendoscopy. The ideal wish of the endoscopist would be to have histological-grade detail down and through the muscularis mucosa. However, current devices do not offer this. OCT is attractive, but current OCT prototypes have several limitations that prevent their use as a stand-alone technique for surveillance. The main clinical advantage of OCT is the ability to stage mucosal disease, as a means of identifying those patients where dysplasia and intramucosal cancer does not penetrate the muscularis mucosa into the submucosa, and therefore would be ideal for curative endoscopic therapy. Although it has the potential of yielding histological details, current OCT prototypes lack sufficient resolution to be considered seriously for real-time endoscopy. Additionally, OCT will only be applicable for viewing small areas of the GI tract.

Within the realm of fluorescence, confocal microendoscopy is a niche area that offers subsurface tomographic 3D reconstructed images of tissue fluorescence and microstructural information. In the future, the use of novel molecularly targeted nanoparticle-based contrast agents may further expand the potential of this emerging technology.

All point spectroscopic techniques, as well as magnification endoscopy, are inherently limited by the small tissue area they sample. However, they contain more detailed information about tissue than any imaging system, which may translate into more accurate tissue differentiation. Rather than competing with an imaging system, the "best" instrument for surveillance may combine imaging and spectroscopy. For instance, a lesion could be detected by fluorescence imaging and its dysplastic nature characterized by Raman spectroscopy or OCT or CFM microprobes. However, in this era of cost containment, such an approach may be prohibitive. Moreover, all of these expensive optical modalities will need to be compared against cheaper and equally promising alternatives, such as chromoendoscopy, for which the dye is inexpensive and colonoscopes are readily available.

The least reported method to date is the use of immune-related fluorescence contrast agents. A limited number of *ex vivo* studies have demonstrated relative GI tumor selectivity with highly fluorescent conjugated antibodies to well-known tumor-associated biomarkers. Such contrast agents have also been evaluated in a very limited number of patients with encouraging enhancement of tumor contrast. However, several important technical issues need to be resolved. This technology also offers the means of aiding our fundamental understanding of disease processes in the GI tract on a molecular level. It is conceivable that in the future, molecular-targeted fluorescence endoscopic imaging will allow earlier detection and characterization of GI disease, and may offer *in vivo* noninvasive monitoring of the functions of a variety of proteins as well as assessment of treatment effects.

"Optical biopsy" refers to tissue diagnosis based on *in situ* optical measurements, which would eliminate the need for tissue removal. The above-mentioned optical techniques are striving toward this goal, but none are likely to replace conventional biopsy and histopathological interpretation in the near future. Although they demonstrate potential for better diagnosis, these modalities are still in their infancy, with future technological refinement and large-scale clinical trials needed to assess their utility and limitations. To date, no commercial systems have withstood the test of comparative clinical trials. Ultimately, whether these optical techniques will become part of standard clinical endoscopic practice or remain on the sidelines can be summed up in two questions: how much better will they perform and at what cost?

## References

1. Lambert, R., Jeannerod, M., et al. (2004). Eyes wide shut. *Endoscopy* **36(8)**, 723-725.
2. DaCosta, R., Lilge, L., et al. (1997). Confocal fluorescence microscopy/macroscopy and microspectrofluorimetry analysis of human colorectal tissues. *Journal of Analytical Morphology: Cell Vision* **4**, 24-29.
3. Zonios, G. I., Cothren, R.M., et al. (1996). Morphological model of human colon tissue fluorescence. *IEEE Trans Biomed Eng* **43(2)**, 113-122.
4. Marchesini, R., Pignoli, E., et al. (1994). Ex vivo optical properties of human colon tissue. *Lasers Surg Med* **15(4)**, 351-357.
5. Cothren, R. M., Richards-Kortum, R., et al. (1990). "Gastrointestinal tissue diagnosis by laser-induced fluorescence spectroscopy at endoscopy. *Gastrointest Endosc* **36(2)**, 105-111.
6. Drezek, R., Brookner, C., et al. (2001). Autofluorescence microscopy of fresh cervical-tissue sections reveals alterations in tissue biochemistry with dysplasia. *Photochem Photobiol* **73(6)**, 636-641.
7. DaCosta, R.S., Andersson, H., et al. (2003). Molecular fluorescence excitation-emission matrices relevant to tissue spectroscopy. *Photochem Photobiol* **78(4),** 384-392.
8. Krammer, B. and Uberriegler, K. (1996). In-vitro investigation of ALA-induced protoporphyrin IX. *J Photochem Photobiol B* **36(2)**, 121-126.
9. Kennedy, J. C., Marcus, S.L., et al. (1996). Photodynamic therapy (PDT) and photodiagnosis (PD) using endogenous photosensitization induced by 5-aminolevulinic acid (ALA): mechanisms and clinical results. *J Clin Laser Med Surg* **14(5)**, 289-304.
10. Baumgartner, R., Huber, R.M., et al. (1996). Inhalation of 5-aminolevulinic acid: a new technique for fluorescence detection of early stage lung cancer. *J Photochem Photobiol B* **36(2)**, 169-174.
11. Leveckis, J., Burn, J.L., et al. (1994). Kinetics of endogenous protoporphyrin IX induction by aminolevulinic acid: preliminary studies in the bladder. *J Urol* **152(2 Pt 1)**, 550-553.
12. Loh, C. S., Vernon, D., et al. (1993). Endogenous porphyrin distribution induced by 5-aminolaevulinic acid in the tissue layers of the gastrointestinal tract. *J Photochem Photobiol B* **20(1)**, 47-54.
13. Peng, Q., Berg, K., et al. (1997). 5-Aminolevulinic acid-based photodynamic therapy: principles and experimental research. *Photochem Photobiol* **65(2)**, 235-251.
14. Battle, A. (1993). Porphyrins, porphyrias, cancer and photodynamic therapy: a model of carcinogenesis. ***J. Photochem. Photobiol B*** **20**, 5-22.
15. Rick, K., Sroka, R., et al. (1997). Pharmacokinetics of 5-aminolevulinic acid-induced protoporphyrin IX in skin and blood. *J Photochem Photobiol B* **40(3)**, 313-319.
16. Webber, J., Kessel, D., et al. (1997). Side effects and photosensitization of human tissues after aminolevulinic acid. *J Surg Res* **68(1)**, 31-37.
17. Lange, N., Jichlinski, P., et al. (1999). Photodetection of early human bladder cancer based on the fluorescence of 5-aminolaevulinic acid hexylester-induced protoporphyrin IX: a pilot study. *Br J Cancer* **80(1-2)**, 185-193.
18. Fijan, S., Honigsmann, H., et al. (1995). Photodynamic therapy of epithelial skin tumours using delta-aminolaevulinic acid and desferrioxamine. *Br J Dermatol* **133(2)**, 282-238.
19. Messmann, H., Knuchel, R., et al. (1999). Endoscopic fluorescence detection of dysplasia in patients with Barrett's esophagus, ulcerative colitis, or adenomatous polyps after 5-aminolevulinic acid-induced protoporphyrin IX sensitization. *Gastrointest Endosc* **49(1)**, 97-101.
20. Kapadia, C.R., Cutruzzola, F.W., et al. (1990). Laser-induced fluorescence spectroscopy of human colonic mucosa. Detection of adenomatous transformation. *Gastroenterology* **99(1)**, 150-157.
21. Richards-Kortum, R., Rava, R.P., et al. (1991). Spectroscopic diagnosis of colonic dysplasia. *Photochem Photobiol* **53(6)**, 777-786.
22. Schomacker, K.T., Frisoli, J.K., et al. (1992). Ultraviolet laser-induced fluorescence of colonic tissue: basic biology and diagnostic potential. *Lasers Surg Med* **12(1)**, 63-78.
23. Cothren, R., Sivak, M., et al. (1996). Detection of dysplasia at colonoscopy using laser-induced fluorescence: A blinded study. *Gastrointestinal Endoscopy* **44,** 168-176.
24. Panjehpour, M., Overholt, B.F., et al. (1995). Spectroscopic diagnosis of esophageal cancer: new classification model, improved measurement system. *Gastrointest Endosc* **41(6)**, 577-581.
25. Panjehpour, M., Overholt, B.F., et al. (1996). Endoscopic fluorescence detection of high-grade dysplasia in Barrett's esophagus. *Gastroenterology* **111(1)**, 93-101.
26. Bourg-Heckly, G., Blais, J., et al. (2000). Endoscopic ultraviolet-induced autofluorescence spectroscopy of the esophagus: tissue characterization and potential for early cancer diagnosis. *Endoscopy* **32(10)**, 756-765.
27. Mycek, M.A., Schomacker, K.T., et al. (1998). "Colonic polyp differentiation using time-resolved autofluorescence spectroscopy. *Gastrointest Endosc* **48(4)**, 390-394.
28. Pfefer, T.J., Paithankar, D.Y., et al. (2003). Temporally and spectrally resolved fluorescence spectroscopy for the detection of high grade dysplasia in Barrett's esophagus. *Lasers Surg Med* **32(1)**, 10-16.
29. Georgakoudi, I., Jacobson, B.C., et al. (2001). Fluorescence, reflectance, and light-scattering spectroscopy for evaluating dysplasia in patients with Barrett's esophagus. *Gastroenterology* **120(7)**, 1620-1629.
30. Harries, M.L., Lam, S., et al. (1995). Diagnostic imaging of the larynx: autofluorescence of laryngeal tumours using the helium-cadmium laser. *J Laryngol Otol* **109(2)**, 108-110.

31. Lam, S., MacAulay, C., et al. (2000). Detection and localization of early lung cancer by fluorescence bronchoscopy. *Cancer* **89(11 Suppl)**, 2468-2473.
32. Wagnieres, G.A., Star,W.M., et al. (1998). "In vivo fluorescence spectroscopy and imaging for oncological applications. *Photochem Photobiol* **68(5)**, 603-632.
33. Haringsma, J., Tytgat, G.N., et al. (2001). Autofluorescence endoscopy: feasibility of detection of GI neoplasms unapparent to white light endoscopy with an evolving technology. *Gastrointest Endosc* **53(6)**, 642-650.
34. DaCosta, R.S., Wilson, B.C., et al. (2000). Light-induced fluorescence endoscopy of the gastrointestinal tract. *Gastrointest Endosc Clin N Am* **10(1)**, 37-69, vi.
35. Yano, H., Iishi, H., Tatsuta, M. (1996). Diagnosis of early gastric cancers by endoscopic autofluorescence imaging system. *Endoscopy* **28**, S29.
36. Zanati, S., Marcon, N., et al. (In Press). Onco-Life fluorescnece imaging during colonoscopy assists in the differentiation of adenomatous and hyperplastic polyps and improves the detection rate of dysplastic lesions in the colon.
37. Kara, M.A., DaCosta, R.S., et al. (2007). Characterization of tissue autofluorescence in Barrett's esophagus by confocal fluorescence microscopy. *Dis Esophagus* **20(2)**, 141-150.
38. Kara, M.A., Peters, F.P., et al. (2006). Endoscopic video-autofluorescence imaging followed by narrow band imaging for detecting early neoplasia in Barrett's esophagus. *Gastrointest Endosc* **64(2)**, 176-185.
39. Messmann, H., Knuchel, R., et al. (1998). Photodynamic diagnosis of gastrointestinal precancerous lesions after sensitization with 5-aminolevulinic acid. A pilot study. *Dtsch Med Wochenschr* **123(17)**, 515-521.
40. Bird, R.P. (1995). Further investigation of the effect of cholic acid on the induction, growth characteristics and stability of aberrant crypt foci in rat colon. *Cancer Lett* **88(2)**, 201-209.
41. Takayama, T., Katsuki, S., et al. (1998). Aberrant crypt foci of the colon as precursors of adenoma and cancer. *N Engl J Med* **339(18)**, 1277-1284.
42. Riddell, R.H., Goldman, H., et al. (1983). Dysplasia in inflammatory bowel disease: standardized classification with provisional clinical applications. *Hum Pathol* **14(11)**, 931-968.
43. Messmann, H., Szeimies, R.M., et al. (1997). Enhanced effectiveness of photodynamic therapy with laser light fractionation in patients with esophageal cancer. *Endoscopy* **29(4)**, 275-280.
44. van den Boogert, J., Houtsmuller, A.B., et al. (1999). Kinetics, localization, and mechanism of 5-aminolevulinic acid-induced porphyrin accumulation in normal and Barrett's-like rat esophagus. *Lasers Surg Med* **24(1)**, 3-13.
45. Ortner, M.A.,Ebert, B., et al. (2003). Time gated fluorescence spectroscopy in Barrett's oesophagus." *Gut* **52(1)**, 28-33.
46. Brand, S., Wang, T.D., et al. (2002). Detection of high-grade dysplasia in Barrett's esophagus by spectroscopy measurement of 5-aminolevulinic acid-induced protoporphyrin IX fluorescence. *Gastrointest Endosc* **56(4)**, 479-487.
47. DaCosta, R., Wilson, B., et al. (In Press). Dosage and timing effects of 5-aminolevulinic aAcid (ALA) on induced protoporphyrin IX (PpIX) fluorescence in patients with Barrett's esophagus. *Gastrointest Endosc*.
48. Kriegmair, M., Baumgartner, R., et al. (1994). Fluorescence photodetection of neoplastic urothelial lesions following intravesical instillation of 5-aminolevulinic acid. *Urology* **44(6)**, 836-841.
49. Leunig, A., Rick, K., et al. (1996). Fluorescence imaging and spectroscopy of 5-aminolevulinic acid induced protoporphyrin IX for the detection of neoplastic lesions in the oral cavity. *Am J Surg* **172(6)**, 674-677.
50. Stummer, W., Stocker, S., et al. (1998). In vitro and in vivo porphyrin accumulation by C6 glioma cells after exposure to 5-aminolevulinic acid. *J Photochem Photobiol B* **45(2-3)**, 160-169.
51. Marcus, S.L., Sobel, R.S., et al. (1996). Photodynamic therapy (PDT) and photodiagnosis (PD) using endogenous photosensitization induced by 5-aminolevulinic acid (ALA): current clinical and development status. *J Clin Laser Med Surg* **14(2)**, 59-66.
52. Shim, M.G. and Wilson, B.C. (1996). The effects of ex vivo handling procedures on the near-infrared Raman spectra of normal mammalian tissues. *Photochem Photobiol* **63(5)**, 662-671.
53. Shim, M.G., Song, L.M., et al. (2000). In vivo near-infrared Raman spectroscopy: demonstration of feasibility during clinical gastrointestinal endoscopy. *Photochem Photobiol* **72(1)**, 146-150.
54. Wong Kee Song, L., Shim, M., et al. (2001). "In vivo endoscopic Raman spectroscopy for the differentiation of dysplasia (low-grade versus high-grade) within Barrett's esophagus. *Gastrointest Endosc* **53(5)**, AB109.
55. Wong Kee Song, L., Molckovsky, A., et al. (2002). Raman spectroscopy for in vivo classification of Barrett's tissue. *Gastroenterology* **122(4 Suppl.)** A288.
56. Molckovsky, A., Song, L.M., et al. (2003). Diagnostic potential of near-infrared Raman spectroscopy in the colon: differentiating adenomatous from hyperplastic polyps. *Gastrointest Endosc* **57(3)**, 396-402.
57. Nie, S. and Emory, S.R. (1997). Probing Single Molecules and Single Nanoparticles by Surface-Enhanced Raman Scattering." *Science* **275(5303)**, 1102-1106.
58. Shetty, G., Kendall, C., et al. (2006). Raman spectroscopy: elucidation of biochemical changes in carcinogenesis of oesophagus. *Br J Cancer* **94(10)**, 1460-1464.
59. Bohorfoush, A. G. (1996). Tissue spectroscopy for gastrointestinal diseases. *Endoscopy* **28(4)**, 372-380.
60. Wallace, M. B., L. T. Perelman, et al. (2000). "Endoscopic detection of dysplasia in patients with Barrett's esophagus using light-scattering spectroscopy. *Gastroenterology* **119(3)**, 677-682.
61. Backman, V., Wallace, M.B., et al. (2000). Detection of preinvasive cancer cells. *Nature* **406(6791)**, 35-36.
62. Gurjar, R. S., Backman, V., et al. (2001). Imaging human epithelial properties with polarized light-scattering spectroscopy. *Nat Med* **7(11)**, 1245-1248.
63. Lovat, L.B., Johnson, K., et al. (2006). Elastic scattering spectroscopy accurately detects high grade dysplasia and cancer in Barrett's oesophagus. *Gut* **55(8)**, 1078-1083.
64. Dhar, A., Johnson, K.S., et al. (2006). Elastic scattering spectroscopy for the diagnosis of colonic lesions: initial results of a novel optical biopsy technique. *Gastrointest Endosc* **63(2)**, 257-261.
65. Reilly, R.M. (1991). Radioimmunotherapy of malignancies. *Clin Pharm* **10(5)**, 359-375.
66. Ballou, B., Fisher, G.W., et al. (1997). Tumor detection and visualization using cyanine fluorochrome-labeled antibodies. *Biotechnol Prog* **13(5)**, 649-658.
67. Haringsma, J. and Tytgat, G.N. (1999). Fluorescence and autofluorescence. *Baillieres Best Pract Res Clin Gastroenterol* **13(1)**, 1-10.
68. Pelegrin, A., Folli, S., et al. (1991). Antibody-fluorescein conjugates for photoimmunodiagnosis of human colon carcinoma in nude mice. *Cancer* **67(10)**, 2529-2537.
69. Folli, S., Westermann, P., et al. (1994). Antibody-indocyanin conjugates for immunophotodetection of human squamous cell carcinoma in nude mice. *Cancer Res* **54(10)**, 2643-2649.
70. Kusaka, Y., Ito, S., et al. (2000). Vital immunostaining of human gastric and colorectal cancers grafted into nude mice: a preclinical assessment of a potential adjunct to videoendoscopy. *J Gastroenterol* **35(10)**, 748-752.
71. Nussbaum, S. and Roth, H.J. (2000). Human anti-mouse antibodies: pitfalls in tumor marker measurement and strategies for enhanced assay robustness; including results with Elecsys CEA. *Anticancer Res* **20(6D)**, 5249-5252.
72. Ramjiawan, B., Maiti, P., et al. (2000). Noninvasive localization of tumors by immunofluorescence imaging using a single chain Fv fragment of a human monoclonal antibody with broad cancer specificity. *Cancer* **89(5)**, 1134-1144.
73. Yokota, T., Milenic, D.E., et al. (1992). Rapid tumor penetration of a single-chain Fv and comparison with other immunoglobulin forms. *Cancer Res* **52(12)**, 3402-2408.
74. Weissleder, R., Tung, C.H., et al. (1999). In vivo imaging of tumors with protease-activated near-infrared fluorescent probes. *Nat Biotechnol* **17(4)**, 375-378.
75. Marten, K., C. Bremer, et al. (2002). Detection of dysplastic intestinal adenomas using enzyme-sensing molecular beacons in mice." *Gastroenterology* **122(2)**, 406-414.
76. Kelly, K., Alencar, H., et al. (2004). Detection of invasive colon cancer using a novel, targeted, library-derived fluorescent peptide. *Cancer Res* **64(17)**, 6247-6251.
77. Tang, Y., Yang, S., et al. (2007). Construction and evaluation of the tumor imaging properties of 123I-labeled recombinant and enzymatically-generated Fab fragments of the TAG-72 monoclonal antibody CC49. *Bioconjugate Chemistry,* **18(3).** 677-684, DOI. 10.1021/bc060260r S1043-1802(06)00260-6
78. MacKinnon, N., Stange, U., et al. (2005). Spectrally programmable light engine for in vitro or in vivo molecular imaging and spectroscopy. *Appl Opt* **44(11)**, 2033-2040.
79. Maunoury, V., Mordon, S., et al. (2000). "Early vascular changes in Crohn's disease: an endoscopic fluorescence study. *Endoscopy* **32(9)**, 700-705.
80. Muguruma, N., Ito, S., et al. (1998). Antibodies labeled with fluorescence-agent excitable by infrared rays. *J Gastroenterol* **33(4)**, 467-471.
81. Bando, T., Muguruma, N., et al. (2002). Basic studies on a labeled anti-mucin antibody detectable by infrared-fluorescence endoscopy. *J Gastroenterol* **37,** 260-269.
82. Hayashi, S., Muguruma, N., et al. (1999). Vital immunohistochemical staining for a novel method of diagnosing micro-cancer. Examination of immunohistochemical staining of non-fixed fresh tissue. *J Med Invest* **46(3-4)**, 178-185.
83. Ito, S., Muguruma, N., et al. (2001). Detection of human gastric cancer in resected specimens using a novel infrared fluorescent anti-human carcinoembryonic antigen antibody with an infrared fluorescence endoscope in vitro. *Endoscopy* **33(10)**, 849-853.
84. Tatsuta, M., Iishi, H., et al. (1989). Diagnosis of gastric cancers with fluorescein-labeled monoclonal antibodies to carcinoembryonic antigen. *Lasers Surg Med* **9(4)**, 422-426.
85. Keller, R., Winde, G., et al. (1998). Immunoscopy--a technique combining endoscopy and immunofluorescence for diagnosis of colorectal carcinoma. *Gastrointest Endosc* **47(2)**, 154-161.
86. Folli, S., Wagnieres, G., et al. (1992). Immunophotodiagnosis of colon carcinomas in patients injected with fluoresceinated chimeric antibodies against carcinoembryonic antigen. *Proc Natl Acad Sci U S A* **89(17)**, 7973-7977.
87. Keller, R., Winde, G., et al. (2002). Fluorescence endoscopy using a fluorescein-labeled monoclonal antibody against carcinoembryonic antigen in patients with colorectal carcinoma and adenoma. *Endoscopy* **34(10)**, 801-807.
88. Krishnadath, K.K., Reid, B.J., et al. (2001). Biomarkers in Barrett esophagus. *Mayo Clin Proc* **76(4)**, 438-446.
89. Srivastava, S., Verma, M., et al. (2001). Biomarkers for early detection of colon cancer. *Clin Cancer Res* **7(5)**, 1118-1126.
90. Josephson, L., Kircher, M.F., et al. (2002). "Near-infrared fluorescent nanoparticles as combined MR/optical imaging probes. *Bioconjug Chem* **13(3)**, 554-560.
91. Horisberger, M. (1981). Colloidal gold: a cytochemical marker for light and fluorescent microscopy and for transmission and scanning electron microscopy. *Scan Electron Microsc* **(Pt 2)**, 9-31.

92. Vo-Dinh, T., Panjehpour, M., et al. (1995). In vivo cancer diagnosis of the esophagus using differential normalized fluorescence (DNF) indices. *Lasers Surg Med* **16(1)**, 41-47.

93. Hanlon, E.B., ***et al.*** (2000) Prospects for *in vivo* Raman spectroscopy. *Phys Med Biol* **45**, R1-R59.
